# Approximate Entropy and Sample Entropy: A Comprehensive Tutorial

**DOI:** 10.3390/e21060541

**Published:** 2019-05-28

**Authors:** Alfonso Delgado-Bonal, Alexander Marshak

**Affiliations:** 1 NASA Goddard Space Flight Center, Greenbelt, MD 20771, USA; 2 Universities Space Research Association, Columbia, MD 21046, USA

**Keywords:** approximate entropy, sample entropy, information theory, chaos theory

## Abstract

Approximate Entropy and Sample Entropy are two algorithms for determining the regularity of series of data based on the existence of patterns. Despite their similarities, the theoretical ideas behind those techniques are different but usually ignored. This paper aims to be a complete guideline of the theory and application of the algorithms, intended to explain their characteristics in detail to researchers from different fields. While initially developed for physiological applications, both algorithms have been used in other fields such as medicine, telecommunications, economics or Earth sciences. In this paper, we explain the theoretical aspects involving Information Theory and Chaos Theory, provide simple source codes for their computation, and illustrate the techniques with a step by step example of how to use the algorithms properly. This paper is not intended to be an exhaustive review of all previous applications of the algorithms but rather a comprehensive tutorial where no previous knowledge is required to understand the methodology.

## 1. Introduction

The objective of approximate entropy (ApEn) and sample entropy (SampEn) is to estimate the randomness of a series of data without any previous knowledge about the source generating the dataset. Therefore, their applicability is limitless, and those algorithms have been used in a wide variety of research fields. Unfortunately, due to the simplicity of the algorithms sometimes the theory behind them is unknown by the researchers, limiting the possibilities to further improve the field or gain a better understanding of what these techniques are measuring.

To fully understand the algorithms, it is necessary to understand the theoretical foundations upon which they are based and what are they trying to measure. Section 2.1 makes an introduction to the concept of randomness based on the analysis of patterns within a dataset, giving an idea of the goal pursued by ApEn and SampEn: to determine how often different patterns of data are found in the dataset.

To achieve that goal, a mathematical measure of the level of randomness is required. That metric is based on the concept of entropy from information theory (IT), a magnitude that quantifies the uncertainty of a measure. In Section 2.2 we provide an introduction to the field and provide the reader with the necessary concepts to understand the rest of the paper. Once we know how to measure information, in Section 2.3 we provide a short description of the ideas from chaos theory that led to the definition of approximate entropy.

The connection between information content and randomness provides a way to quantify the level of randomness of a dataset in a purely mathematical way, without assuming any underlying model or hypothesis about the process generating the data. In Section 3.1 we analyze the definition and properties of approximate entropy, and in Section 3.2 we introduce cross-ApEn, useful when looking for patterns from one dataset into another.

Due to its theoretical definition, ApEn is a biased statistic; in an attempt to define an unbiased statistic, sample entropy was presented as an alternative regularity statistic with slightly different foundations, described in Section 3.3. Its extension to two datasets, cross-SampEn, is summarized in Section 3.4.

Once the theoretical framework is explained, we illustrate how to use it with a step by step example. In Section 4.1 we analyze the temperature record of a location in New York City during the last 150 years, providing detailed explanations of each step and a code in R programming language to calculate ApEn in Appendix A. The same dataset is used in Section 4.2 to calculate SampEn, and similarly, a code in R is presented in Appendix B.

Finally, a comparison between the two algorithms is carried out in Section 4.3, providing some clarifications about how to use them, and the conclusions are summarized in Section 5.

## 2. Information and Complexity

### 2.1. Regularity Statistics

In many situations the use of the word random contrasts with the mathematical idea of the randomness of a number. In the words of the mathematician Gregory J. Chaitin [1]: “*Although randomness can be precisely defined and can even be measured, a given number cannot be proved to be random. This enigma establishes a limit to what is possible in mathematics.*”

Randomness can be adequately defined and is usually studied probabilistically, analyzing the frequencies of occurrence of different digits in a series and using measures such as mean, variance, asymmetry, kurtosis, etc. However, the analysis of the randomness of a series from this perspective presents some problems that classical methods cannot overcome.

To study the concept of randomness of a series, Chaitin proposed the experiment of flipping a coin 20 times and write down a one if it comes heads and a zero if it comes tails. From a probabilistic perspective the experiment could produce the next two series with the same probability (one over $2^{20}$), thus considering both series as random (note that the original series B in Chaitin’s example is 01101100110111100010; However, that series has a different mean than series A. To emphasize the fact that the two series are indistinguishable from the point of view of mean, variance, etc., we have modified series B to have ten ones and ten zeros, like series A):

A: 01010101010101010101
B: 01101000110111100010

While it is true that the two series have been produced randomly with the same probability and have the same values of mean and variance, it is easy to continue the pattern described in series A, but we would have to work harder to be able to predict the next number in series B. Series A is constructed by alternating zeros and ones, while series B does not have a clear pattern. The analysis of randomness is, therefore, an essential limitation of the methods of moment statistics and cannot be used to guarantee the randomness of a series mathematically. If we shuffle any of the previous two series we would obtain the same values using moment statistics since the calculations are independent of the organization, their terms are of the form $\sum_{i}{\left(x_{i}-\bar{x}\right)}^{a}$, where *a* is the order of the calculated moment.

The origin of this limitation lies in the fact that the classical probabilistic approach of analyzing the moments of different orders is not analyzing the randomness of a series but the randomness of the generating process of the series. A higher frequency of a number in a series, an asymmetry of the distribution, or any other similar measure, are tools to study the generating process of the data, and the order of their generation does not have any influence. Consequently, as Chaitin claimed, the use of those methods cannot prove the randomness of a given number, just because the underlying idea of probability is to analyze the generation method.

The analysis of the randomness of a series has its roots in information theory and the concept of entropy. However, it is necessary to make a couple of clarifications to avoid misunderstandings later. First of all, it is essential to make clear that information theory (IT) is a branch of mathematics. Claude E. Shannon published the basics of IT in an article entitled “A Mathematical Theory of Communication” [2], and those ideas have been used in different fields since then, from biology to economics and physics.

The second clarification is, precisely, concerning physics. It is not strange to find sources saying that the base of information theory is statistical mechanics. In many occasions, there is an emphasis in pointing out that the mathematical apparatus used in information theory is similar to the one used by Boltzmann in his kinetic theory of gases and, in general, in statistical mechanics. While the mathematical techniques are similar, information theory is entirely independent of physics; it is a mathematical theory.

The last clarification was necessary because the fundamental concept in information theory is called entropy. In physics, entropy is a perfectly defined magnitude whose origins lie in thermodynamics and statistical mechanics, defined by Clausius and Boltzmann respectively. The concept of entropy in information theory did not originate following any of those ideas, and the choice of the word entropy for its fundamental definition was either a good joke or an extraordinary intuition (if we believe the story, Shannon thought about naming his new concept “information” or “uncertainty”, but those names had already been used extensively prior to his formulation. Then John von Neuman suggested him to use the name entropy because in the statistical mechanics version the equations of entropy have the same isomorphism and, on the other hand, nobody really knows what entropy is so he would have an advantage and would win any dispute that might arise) [3,4]. The positive relationship between physics and information theory is a fascinating field of research, but it is not the objective of this work. Some comments will be made in the following sections when necessary, but it is important to note once again that the fundamentals of information theory are purely mathematical.

In essence, since entropy quantifies the amount of information, it also measures the degree of randomness in the system. It would be desirable that one of the characteristics when measuring the randomness of a series were to establish a hierarchy of degrees of randomness, a quantification of the level of randomness so that the question would not be whether a data series is random or not, but how random it is. There will be a situation with maximum randomness, totally unpredictable, but there will also be other situations with different degrees of randomness so we should be able to order the data series according to their complexity. A simple (non-complex) data series would have obvious patterns, such as the above-mentioned series A, while for a complex series those patterns would be completely non-existent.

This paper is devoted to explaining that approach, focusing on analyzing the randomness of the series by studying the patterns of numbers using a fundamental mathematical formulation without assuming any model. Information theory provides us with the necessary tools to carry out this task, and in particular, this paper follows the approach of Chaitin [1] and Kolmogorov [5] in defining complexity in the algorithmic information theory. An excellent review of the methods of information theory is presented in [6], useful for learning the basics of information theory or for researchers who want to learn new methods to obtain information from limited and noisy data.

Measuring information is, in fact, the essential contribution of information theory. This theory answers positively and mathematically the question “can we objectively measure information?” through the concept of entropy. Once we know how to measure information, it is possible to define randomness, an idea which falls within IT in the coding theory.

The underlying idea is the following. Imagine the days when the telegraph was in use, and imagine two researchers, one who reads the temperatures in New York and sends telegrams to his friend in Chicago, an expert meteorologist who wants to get data from the previous year for his research. Coding theory deals with the information content of the telegrams and the minimum length required to send the message from one place to another.

Suppose that the person in New York writes down a “1” if the temperature rises and a “0” if it goes down, writing a number per hour and making the yearly series extremely long. If the weather behaved like a perfect sinusoidal series, the telegram would consist of the series of numbers:

‘The values were 01010101010101010101010101010101010101010101010101…’

However, the person in New York can simplify the telegram (and save money with it, remember that the cost depended on the number of words) by telling his friend:

‘The values were 01 repeated 4380 times.’

The essential point to note is that the information is the same in the two messages; however, the length of the code is minimal in the second case, making it optimal (and cheaper).

On the contrary, suppose the weather does not behave in such a good way, and the sequence of numbers makes no sense to the person in New York. There is no other option but to send the complete list of numbers in a telegram of the form:

‘The values were 01101101000100111010101010100101010110101011111010…’

As there is no discernible pattern in those data, there is no way to reduce the telegram length, thus paying a higher cost for it. The essential point here is that we cannot compress a sequence of totally random numbers into a shorter message, which offers a way to define the total randomness of a series as a message which requires the maximum length to be transmitted.

Once randomness is defined in this way, depending on its content of information and the length of the code necessary to send the message, we can consider how to measure it in different degrees. As we have seen in the previous example, the existence of patterns within a series is the core of the definition of randomness, so it is appropriate to establish a hierarchy of randomness based on the different patterns and their repetitions. The mathematical formulation to achieve it comes from another branch of mathematics called Chaos Theory. Following those ideas, Pincus [7] described the methodology of ApEn to measure the randomness of limited and noisy data, applying it initially to different series of clinical and physiological data. The technique was expanded to other fields and was found useful to distinguish order in series generated by stochastic and deterministic movements.

### 2.2. Information Theory

In this section we define and summarize those concepts of IT necessary to understand the logic behind ApEn, focusing on discrete variables. It is not intended to be a complete review of the subject or to give demonstrations or new methods to analyze data. The reader familiar with IT can skip this section, and those interested in having a broader idea on the subject can consult excellent general books [8] or great reviews [6].

Originally, IT was designed as a theory to analyze the process of sending messages through a noisy channel and understanding how to reconstruct the message with a low probability of error. As stated before, one of the ideas behind IT is the fact that the information in a message can be measured quantitatively. When studying how to measure this quantity, two researchers from Bell laboratories [9,10] concluded that the logarithmic function is the natural choice to measure the amount of information due to the properties of this function. The analysis of communications by telegraph led them to present a formula to measure information of the type H=nlogS, where *S* is the number of possible symbols (we will call the possible number of symbols *alphabet* later on) and *n* the number of them transmitted.

Those ideas were correct but limited to situations in which all events have the same probability, such as flipping a coin or rolling a dice. Shannon extended this formulation to bypass that restriction, proposing entropy as the measure of information of a single random variable. The idea of a random variable in this context refers to the probabilistic interpretation: each of the possible outcomes of the variable has a defined probability of occurrence and the value associated with the outcome is aleatory. If *X* can take the values $\left\{x_{1},\ldots ,x_{n}\right\}$ and p(x) is the probability associated with those values given x∈X, entropy is defined as:

(1) $$H\left(X\right)=-\sum_{x\in X}p\left(x\right)\log p\left(x\right).$$

The logarithm of base 2 is the usual choice but any other base can be used since $H_{b}\left(X\right)=\log_{b}\left(a\right)\left[H_{a}\left(X\right)\right]$. Also, by convention, the entropy H(0)=0 since $\lim_{p\to 0}p\cdot \log_{2}\left(p\right)=0$. This magnitude determines the average or expected information of an event, determined as the mathematical expectation $E[\log\left(\frac{1}{p(X)}\right)]$. It is important for understanding the following sections to realize that entropy is based on the probability of occurrence of each symbol, i.e., entropy is not a function of the values of the series themselves but a function of their probabilities. In general, the researcher ignores the real probability distribution of each symbol and uses the frequency of occurrence in the data series as an approximation of the real probability.

It is a good idea to interpret the fundamental concepts of IT before continuing with the mathematical description. Following [6]:

- Information is the decrease in ambiguity regarding a phenomenon, an increment to our knowledge when we observe a specific event. Realizations of a random variable with low probability are more surprising and add more information. Imagine a series of data whose values change so little that the next value is practically the same as the previous one. If there is an abrupt change and the value increases drastically (this random variable having a high value as an outcome is an event with low probability), there is a high gain of information. Mathematically, information is proportional to the inverse of the probability function of the event, $\log_{2}\frac{1}{p_{i}}$. Therefore, information is an event which induces rational agents to change their beliefs and expectations about a particular situation, with the restrictions of the new information. In the previous example, very likely events (a flat series of data) give little information to the agents, while low probability outcomes (a sudden rise in the value) give more information that will change their beliefs about the process behind the data.
- Uncertainty is something which is possible but is unknown. It is possible that by throwing a dice we get the number five, but throwing a dice does not imply obtaining the number five. We have uncertainty about the number five, but we would not have it for the number 214 in a six-sided dice because it is an impossible event. In the context of IT, uncertainty is the amount of information we expect an experiment to reveal. If the distribution of probabilities is uniform, as in the case of the dice ($p_{i}=1/n$), there is a maximum uncertainty and the result is more unpredictable. On the other hand, if the probability distribution is more concentrated (a loaded dice with a high probability of obtaining a six), then the result is more predictable and there is a lower uncertainty. Concerning the previous concept, low uncertainty means more information.
- Entropy (H${}_{x}$) is a measure of the amount of uncertainty associated with a variable *X* when only its distribution is known. It is a measure of the amount of information we hope to learn from an outcome (observations), and we can understand it as a measure of uniformity. The more uniform the distribution, the higher the uncertainty and the higher the entropy. This definition is important because entropy will ultimately be used to measure the randomness of a series. The entropy *H* is a function of the probability distribution {$p_{1},p_{2},\ldots$} and not a function of the values of the variable in the particular series {$x_{1},x_{2},\ldots$}.

**Figure 1:**
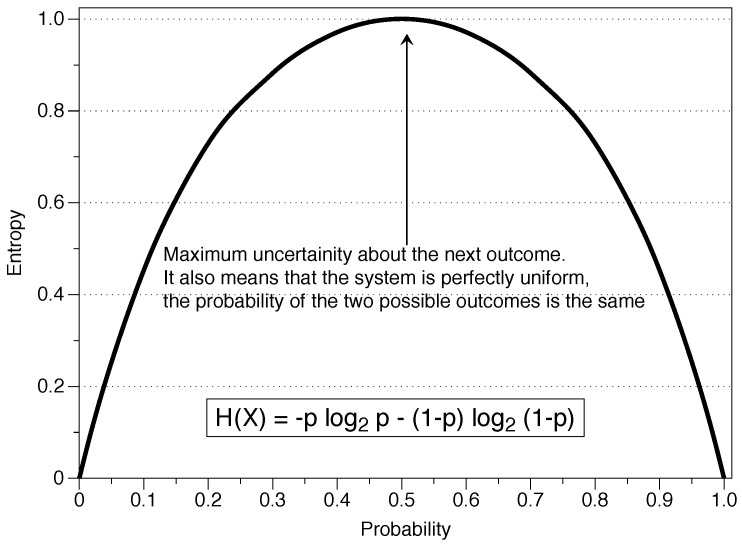
Entropy of a variable *X* which takes the value one with probability *p* and the value zero with probability (1−p).

Information theory and the concept of entropy provide a mathematical way of determining the information contained in a message. This information is independent of the receiver’s knowledge and is determined by the source of information via the probability function of each of the symbols. The result of an experiment contains the amount of information H(p), or stated differently, H(p) is a measure of the uncertainty about a specific event before observing it. As we can see in Figure 1, the entropy depends on the amount of randomness represented by *p*, where the situation p=1/2 is random from a probabilistic perspective and gives a maximal entropy H = 1. With Shannon’s definition, events with high or low probability do not contribute much to the value of the measure, as shown in the graph for values of p=0 or p=1.

By extending the notion of entropy to more than a single random variable we can now define concepts that will be related to other magnitudes in the following sections. Let p(X,Y) be the joint probability distribution of two discrete random variables whose possible realizations are {$x_{1},x_{2},\ldots ,x_{K}$} for *X* and {$y_{1},y_{2},\ldots ,y_{J}$} for *Y*. We define $P\left(X=x_{k}\right)=p_{k}$, $P\left(Y=y_{j}\right)=q_{j}$, $P\left(X=x_{k},Y=y_{j}\right)=w_{kj}$, $P\left(X|Y\right)=P\left(X=x_{k}|Y=y_{j}\right)=p_{k|j}$ and $P\left(Y|X\right)=P\left(Y=y_{j}|X=x_{k}\right)=q_{j|k}$, where it is satisfied that $p_{k}=\sum_{j=1}^{J}w_{kj}$, $q_{j}=\sum_{k=1}^{K}w_{kj}$ and $w_{kj}=q_{j}p_{k|j}=p_{k}q_{j|k}$.

The conditional entropy H(X|Y) measures the total amount of information contained in the variable *X* given the condition that *Y* assumes a certain value:

(2) $$H\left(X|Y\right)=\sum_{k,j}w_{kj}\log\left(\frac{q_{j}}{w_{kj}}\right).$$

The joint entropy of *X* and *Y* is given by:

(3) $$H\left(X,Y\right)=\sum_{k,j}w_{kj}\log\left(\frac{1}{w_{kj}}\right)$$

The chain rule for the entropy relates the two previous quantities:

(4) H(X,Y)=H(Y)+H(X|Y)=H(X)+H(Y|X).

If *X* and *Y* are independent, then H(X,Y)=H(X)+H(Y).

The relative entropy, cross-entropy, or Kullback–Liebler distance function measures the distance between two probability functions *p* and *q*:

(5) $$D\left(p||q\right)=\sum_{k=1}^{K}p_{k}\log\left(p_{k}/q_{k}\right).$$

We can interpret this measure in different ways. It reflects the gain of information resulting from adding the knowledge in *p* relative to *q*, or the gain of information when we learn that the real probability function was *p* instead of *q* as we thought initially. However, it should be noted that it is not a true measure of distance because it is not symmetric D(p||q)≠D(q||p).

Finally, the mutual information between the random variables *X* and *Y* determines the reduction in uncertainty in *X* given our knowledge of *Y*, or the amount of information contained in a random variable about the other:

(6) $$I\left(X;Y\right)=\sum_{k,j}w_{kj}\log\left(\frac{w_{kj}}{p_{k}q_{j}}\right)=D(w_{kj}\left|\right|p_{k}q_{j})=H\left(X\right)-H\left(X|Y\right).$$

If the processes are independent there is no information gain H(X|Y)=H(X) and I(X;Y)=0, being I(X;Y)>0 in any other case. Mutual information is a symmetric measure, I(X;Y)=I(Y;X)=H(X)+H(Y)−H(X,Y), and is intimately related to the likelihood tests (log-likelihood ratio) so that it can be considered a statistic to determine the independence between two variables, having a well specified asymptotic distribution.

Finally, although it is not strictly part of information theory, it is interesting to dedicate a few words to the maximum entropy principle (MEP). MEP tries to show a procedure to estimate the unknown distribution of a random variable with the minimum hypotheses about the underlying likelihood function, making inferences with limited and insufficient data. The idea behind MEP is that on many occasions researchers only have access to the observed moments of the sample (mean, variance, etc.) but do not have the sample itself. In these circumstances, if the researcher makes hypotheses about the distribution of the random variable or about the generating process of that sample, the conclusions may be erroneous. The maximum entropy principle uses only the information available to the researcher (introducing the information as a restriction in an optimization problem) and is, therefore, the best estimate that we can obtain without having more knowledge about the variable.

Jaynes developed the MEP formalism based on the entropy function of Shannon [3,4], and his ideas have been of great importance in the development of information theory. MEP is an efficient way to process information (information processing rule), which means that the total information after using this principle is the same as before using it, i.e., it uses all available information without making any hypothesis. Jaynes’s ideas on probability and inference led him to present the maximum entropy principle as the best way to determine a priori probabilities, since it uses only known information and the irrelevant details are eliminated by averaging over them.

The understanding of entropy as a tool for inference is carried out by comprehending that it is a process of updating the a priori probabilities when we receive new information. In this sense, entropy is intimately related to Bayes’ theorem and, once again, it is only a mathematical tool. The relationship between IT, MEP and Bayesian probability is of great mathematical importance, and the interested reader can find out more about their relationship and implications in Zellner’s texts [11,12,13].

### 2.3. Towards a Measure of Complexity

In the previous section, we defined entropy as a measure to quantify the information of a message which is valid in a general way, independently of the process generating it. A particular kind of processes are those called stochastic. We can understand these processes as a succession of random variables related to each other by a rule of evolution, representing a system which evolves over time. The opposite type of processes can be called deterministic, a system in which there is no randomness in the determination of the following states of the system. In these processes there is no diffusion and the equation of evolution of the probability is given by the evolution operator of Perron–Frobenius in discrete systems or the Liouville equation in continuous systems; if the original distribution is a Dirac delta, a delta is maintained for a deterministic system but not for a stochastic one (Fokker–Planck equation), in which case it widens.

Stochastic processes can be divided into random walks, martingales, Markov processes, etc., which has motivated their extensive use in different fields. On the other hand, Chaos Theory studies the deterministic systems in mathematics, situations in which knowing the initial state of the system accurately we can make an exact prediction of any future state of the system.

The question of evaluating the randomness of a series of data is, therefore, a matter of characterizing the system as stochastic or deterministic. More precisely, to which extent the data behaves as stochastic and how much determinism exists; we would be talking about degrees of randomness. If there is no underlying deterministic process when analyzing a particular series of data, it will imply a verification of the strictest random movement. However, if there are cycles, trends, and patterns, then the data series would not be totally random. It will be necessary to quantify both the totally random situation and the different degrees of randomness in an unequivocal way.

In the particular case of stochastic processes, the interest does not lie in measuring the total entropy but in measuring the entropy of the process, i.e., how the entropy of the series changes over time. If we think of a series of data, our interest would be to measure the increase of information that we obtain when we know a new value. This measure is determined by the entropy rate of the stochastic process {$X_{t}$} with x∈K, where *K* is the alphabet (when the limit exists):

(7) $$H\left(X\right)=\lim_{T\to \infty }\frac{1}{T}H\left(X_{1},X_{2},\ldots ,X_{T}\right).$$

The magnitude H(X) is a measure of the entropy per symbol of the variable $X_{t}$. It gives an idea of the temporal density of the average information of a stochastic process. A conditional amount can be defined as:

(8) $$H^{*}\left(X\right)=\lim_{T\to \infty }H\left(X_{T}|X_{T-1},X_{T-2},\ldots ,X_{1}\right),$$

which determines the entropy of the last random variable conditioned to the previous T−1 values. For completely stochastic independent and identically distributed (i.i.d.) variables process the relation is simply $H\left(N\right)=H^{*}\left(N\right)$, i.e., the entropy rate which is equal to the entropy of each member of the process. Hence, the measurement of the entropy rate offers a way of measuring to what extent a process is stochastic and will ultimately quantify the randomness of a series [8] (p. 75).

We can relate this concept to the previous discussion about the telegraph. The entropy rate is the expected number of bits (measure of entropy when we use the base 2 for the logarithm) per symbol needed to describe the process. If the process is entirely stochastic, then the number of bits is maximum (the entropy rate is maximum). By comparing the maximum entropy rate of our system with the maximum number of bits given an alphabet we can determine the degree of randomness of a series without assuming anything about the data generating process.

The approach followed to present the example of the telegraph is known as coding theory. Within this theory, source theory is the part of information theory which uses the entropy rate defined above. It is possible to understand any process that generates successive messages as a source of information, and the rate of the source of information is related to its redundancy and its level of compression. In the example of the telegraph, one situation was very redundant and could be compressed while the other was incompressible, being utterly random (compress in this context has the same meaning as in computer science when we speak of a compressed file .zip, .tar, .rar, etc.).

A source without memory produces i.i.d. messages. For those sources, the entropy rate is the entropy of each symbol, i.e., the knowledge of the previous values of the series does not give any information to predict the next result, generating a completely random series. The connection between information theory, sending a message through a channel, and statistical experiments was developed by Lindley [14], who understood that a statistical sample could be seen as a noisy message which carries information about a parameter with a specific distribution, therefore talking about information in the experiment instead of information in the message.

The field of information theory which analyzes randomness is known as Algorithmic Information Theory. This field studies the complexity in data series by analyzing its entropy. As explained above, the information content of a series is equivalent to the length of the shortest possible message which represents that series. Kolmogorov’s definition of complexity is the base of the mathematical framework to measure randomness, independently of any physical or philosophical concept: a binary chain is random if the Kolmogorov complexity of that chain is at least the length of the chain. By defining the algorithmic complexity as the length of the shortest program (telegraph) that can print that message, a string is called random if it is incompressible, i.e., its complexity is equal to its length.

The use of the entropy rate to study the complexity of a chain is not limited to stochastic processes. It is possible to describe the structural similarity between different dynamic systems in the same way. In order to do that, Sinai [15] introduced the concept of entropy for dynamic systems that preserve the measurements, giving a generalization of Shannon entropy for dynamic systems, known as Kolmogorov–Sinai entropy (KS) nowadays. For dynamic systems the (algorithmic) complexity and the entropy rate of the trajectories are related in almost all situations via KS(x; T) = H(T), meaning that the complexity is measured via the entropy rate.

As we have seen, in both stochastic and deterministic systems the entropy rate can be related to the randomness of the system. A particular type of systems where KS entropy is of great interest are chaotic systems. In such systems the slightest inaccuracy is amplified over time, although if it is exact it is preserved. To analyze these systems we must consider all the possible states of the dynamic system (the phase space) creating a *F*-dimension partition by dividing it into cells of size $r^{F}$ and measuring the state of the system at constant time intervals τ. Let $p(i_{1},i_{2},\ldots ,i_{d})$ be the joint probability that $\bar{x}\left(t=\tau \right)$ is in cell $i_{1}$, $\bar{x}\left(t=2\tau \right)$ is in cell $i_{2}$, …, $\bar{x}\left(t=d\tau \right)$ is in cell $i_{d}$. The KS entropy is then calculated as:

(9) $$KS=-\lim_{\tau \to 0}\lim_{r\to 0}\lim_{d\to \infty }\frac{1}{d\tau }\sum_{i_{1},\ldots ,i_{d}}p\left(i_{1},\ldots ,i_{d}\right)\times \log p\left(i_{1},\ldots ,i_{d}\right).$$

The joint probability determines the trajectory and the characterization of the system is as follows: KS = 0 for ordered systems, KS is infinite for random systems and KS is constant (≠0) in deterministic chaotic systems. However, we can see in the previous equation how the calculation of the KS entropy involves the limiting case of the time and space partitions tending to zero and an infinite number of points. These restrictions make it possible to determine the KS entropy for well-defined analytical systems but challenging for limited and noisy measurements of a signal represented in a data series.

To overcome those limitations, methods were developed to analyze the KS entropy of a temporal signal, allowing the comparison of models and experimental data. Instead of using Shannon entropy, Grassberger and Procaccia [16] realized that using a Rényi entropy (a generalization of Shannon entropy) of order 2, $K_{2}$, the calculations were suitable for time series. Denoting any coordinate such as *X*, they defined a measure of the information rate generated in a chaotic system for a given data series such as:

(10) $$C_{d}\left(r\right)=\lim_{N\to \infty }\frac{1}{N^{2}}\left[\mathrm{number}\;\mathrm{of}\;\mathrm{pairs}\;\left(n,m\right)\;\mathrm{with}{\left(\sum_{i=1}^{d}{\left|X_{n+i}-X_{m+i}\right|}^{2}\right)}^{1/2}\leq r\right].$$

This magnitude, named correlation integral, is of essential importance; it measures with a tolerance *r* the regularity, or frequency, of patterns similar to a given template of a given length (which will be known as *m* later on). The idea is that to reconstruct the trajectory of a dynamic system we do not need to follow the evolution of all degrees of freedom, since it can be reconstructed with *d* measures (d≥F) of a single coordinate. Basically, by following this procedure we could approximate the entropy of a data series in a simple way:

(11) $$K_{2,d}\left(r\right)=\frac{1}{\tau }\log\frac{C_{d}\left(r\right)}{C_{d+1}\left(r\right)}\;\;\mathrm{and}\;\;\lim_{\begin{array}{c} d\to \infty \\ r\to 0 \end{array}}K_{2,d}\left(r\right)∼K_{2}.$$

Formally, for the analysis of time series the correlation integral is defined as:

(12) $$C\left(r\right)=\lim_{N\to \infty }\frac{1}{N^{2}}\sum_{i,j=1}^{N}\theta (r-|\bar{X_{i}}-\bar{X_{j}}\left|\right),$$

where θ(x) is the Heaviside function. It is interesting to point out that a finite value of the correlation integral does not imply an underlying determinism [7].

Takens changed the Euclidean metric in the previous definition by a measure of the maximum distance (Chebyshev distance), introducing the difference functions (*d* functions) between two vectors [17]. If u={u(1),u(2),…,u(N)} is a series of length *N*, using a non-negative number m≤N to define the length of the blocks x(i)={u(i),u(i+1),…,u(i+m−1)} and x(j)={u(j),u(j+1),…,u(j+m−1)} as sequences of that series, the distance between them is given by:

(13) $$d\left[x\left(i\right),x\left(j\right)\right]=max_{k=1,2,\ldots ,m}(|u\left(i+k-1\right)-u\left(j+k-1\right)|).$$

Eckmann and Ruelle defined later the magnitude [18]:

(14) $$\phi^{m}\left(r\right)={\left(N-m+1\right)}^{-1}\sum_{i=1}^{N-m+1}\log C_{i}^{m}\left(r\right),$$

in order to define an estimation of the entropy (ER entropy) as:

(15) $$E-R\;\mathrm{entropy}=\lim_{r\to 0}\lim_{m\to \infty }\lim_{N\to \infty }\left[\phi^{m}\left(r\right)-\phi^{m-1}\left(r\right)\right].$$

The interpretation is that $\phi^{m}\left(r\right)-\phi^{m-1}\left(r\right)$ is the average over *i* of the logarithm of the probability that |u(j+m)−u(i+m)|≤r, given the condition that the previous values fulfill the condition |u(j+k)−u(i+k)|≤r for k=0,1,…,m−1; i.e., it is a conditional probability.

These equations offer a way to measure the dynamics of a system by calculating the entropy. However, although these ideas are the core for assessing the randomness of a series, they present problems when applied to experimental data series.

## 3. Measuring Randomness

### 3.1. Approximate Entropy

The formulation for the analysis of the complexity of a system was developed to analyze chaotic systems, and the attempts to use those techniques for limited, noisy and stochastically derived time series were not very successful. The algorithm of the correlation integral and the Kolmogorov–Sinai entropy work well for real dynamic systems, but even a small amount of noise makes those algorithms useless, with the values tending to infinity. Besides, a finite correlation dimension value does not guarantee that the process under analysis is deterministic.

KS entropy classifies the deterministic systems depending on the rates of generation of information by determining an entropy rate. However, it is not possible to use the entropy formalism developed in the previous section for statistical calculations. We can see that the formulation involves the calculation of several limits, which even though it is mathematically possible, it represents an unrealistic situation since in real life we do not have an infinite number of points or a noiseless system. Even small amounts of noise (stochastic behavior) make the KS entropy not convergent in many situations.

To compare systems with deterministic and stochastic components, Pincus developed a new statistic for experimental data series [7]. In case of dealing with a purely dynamic system (deterministic) with explicit analytical expressions, the techniques of the previous section are more suitable for its analysis since they reconstruct better the probabilities of the phase space. However, if some stochastic component is present, the ApEn algorithm is more useful since the statistical precision of the other methods is compromised.

ApEn is not intended to be an approximation of the KS entropy but a statistic for the measure of regularity whose foundations are similar and correctly quantifies finite data series; it was devised as a quantification of the rate of regularity in time data series. As stated earlier, Pincus realized that in many cases the direct application of the KS entropy [16,17] was inaccurate or incorrect, and the reason was the existence of a low magnitude of noise and a limited number of data. Let {u(1),u(2),…,u(n)} be a series of data and be {v(1),v(2),…,v(n)} a series generated as white noise. Using the mathematical formulation of the entropy from the previous section, the entropy of {u(1)+v(1),u(2)+v(2),…,u(n)+v(n)} would be infinite due to the stochastic component.

The regularity statistic ApEn was formulated with the same philosophy of KS entropy (to measure the entropy rate of a system) but was built to solve those limitations. The number of points necessary to properly characterize a dynamic system is in the range $\left[10^{d},30^{d}\right]$, where *d* is the dimension of the system. Therefore, a large amount of data is required to fully characterize even low-dimensional systems.

ApEn is a parameter that measures correlation, persistence or regularity in the sense that low ApEn values reflect that the system is very persistent, repetitive and predictive, with apparent patterns that repeat themselves throughout of the series, while high values mean independence between the data, a low number of repeated patterns and randomness. This definition coincides with the intuition that the systems which have more random probability have higher entropy, as seen in the previous sections, being pure stochastic systems those which had a higher entropy rate (maximum number of bits given an alphabet). In binary systems, the maximum value of ApEn would be log2, and a value lower than that would indicate that the series under analysis contains patterns which are repeated and therefore is not totally random.

Heuristically, ApEn (like the entropy defined by Eckmann and Ruelle) measures the logarithmic probability that nearby pattern runs remain close in the next incremental comparison. ApEn quantifies the concept of variable complexity without the difficulties of exact statistics of regularity. The main idea behind ApEn’s development was that it is not an algorithm to entirely determine the dynamics of a system. Instead, it is an appropriate algorithm for classifying systems and studying the evolution of its complexity: it is not necessary to completely reconstruct the dynamics of the system to classify it.

Mathematically, the idea behind Pincus’s work is that if the “joint probability measures for reconstructed dynamics describing each of two systems are different, then their marginal probability distributions on a fixed partition, given by conditional probabilities, are likely different [...]. It is essential to note that marginal probabilities of two processes may be equal while the joint probabilities are quite different. However, if two measures have distinctly different marginal probabilities, then that alone is sufficient to discriminate the measures” [19].

Marginal probabilities are a partial characterization of the process, while the joint probability measure is a mean of assigning a probability to a region of space, used to reconstruct the dynamics of the system entirely. Typically, it takes fewer points (orders of magnitude less) to calculate those marginal probabilities than to reconstruct the process completely. ApEn does not attempt to reconstruct the full dynamics but only discriminate in a statistically valid way, and in return, we can get rid of the limits m→∞ and r→0, making the approach suitable for data analysis. We will see how to choose the values of *m* (embedding dimension) and *r* (scaling parameter or noise filter) to calculate ApEn, but it is appropriate to understand how those changes affect the system.

In the ApEn algorithm, we must select a pair (m,r) as input parameters. Statistically, it would be the equivalent of dividing the space of states into cells of width *r*, to estimate the conditional probabilities of the *m*-th order. In the interpretation given below, *m* is the length of the template (length of the window of the different vector comparisons), and *r* is a de facto noise filter (superposition of noise much smaller in magnitude than *r* barely affects the calculation). Higher *m* and small *r* describe details of sharper (probabilistic) parameters. However, when dealing with stochastic processes, the analysis of conditional probabilities causes large values of *m* or minimal values of *r* to produce statistically deficient estimates. The following example and the example in Section 4.1 will visually illustrate the meaning of these parameters.

Formally, the algorithm for determining the Approximate Entropy of a sequence is as follows (it is also suggested to see the illustrative example in [20]):

Given a sequence of numbers u={u(1),u(2),…,u(N)} of length *N*, a non-negative integer *m*, with m≤N and a positive real number *r*, we define the blocks x(i)={u(i),u(i+1),…,u(i+m−1)} and x(j)={u(j),u(j+1),…,u(j+m−1)}, and calculate the distance between them as $d\left[x\left(i\right),x\left(j\right)\right]=max_{k=1,2,\ldots ,m}(|u\left(i+k-1\right)-u\left(j+k-1\right)|)$. Then we calculate the value $C_{i}^{m}\left(r\right)$ = (number of j≤N−m+1 such that d[x(i),x(j)]≤r)/(N−m+1). The numerator of $C_{i}^{m}$ counts, within the resolution *r*, the number of blocks of consecutive values of length *m* which are similar to a given block.

Computing:

(16) $$\phi^{m}\left(r\right)=\frac{1}{N-m+1}\sum_{i=1}^{N-m+1}\log C_{i}^{m}\left(r\right).$$

We can define ApEn(m,r,N)(u) = $\phi^{m}\left(r\right)-\phi^{m+1}\left(r\right)$, with m≥1 and ApEn(0,r,N)(u) = $-\phi^{1}\left(r\right)$.

ApEn(m,r,N)(u) measures the logarithmic frequency with which blocks of longitude *m* that are close together stay together for the next position, or put differently, the negative value of ApEn is defined as:

– ApEn$\left(m,r,N\right)\left(u\right)=\phi^{m+1}\left(r\right)-\phi^{m}\left(r\right)$ = average over *i* of the logarithm (conditional probability of |u(j+m)−u(i+m)|≤r, if it is verified that |u(j+k)−u(i+k)|≤r for k=0,1,…,m−1).

ApEn(m,r,N) is the statistical estimator of the parameter ApEn(m,r):

(17) $$ApEn\left(m,r\right)=\lim_{N\to \infty }\left[\phi^{m}\left(r\right)-\phi^{m+1}\left(r\right)\right].$$

ApEn(m,r,N) is a family of statistics and the comparisons between systems are intended with fixed values of *m* and *r*, and, if possible, with the same number of observations *N* due to the bias that we will mention later. ApEn “measures the likelihood that runs of patterns that are close for *m* observations remain close on next incremental comparisons. Greater likelihood of remaining close, implying regularity, produces smaller ApEn values, and conversely” [20]. Ultimately, the conditional probabilities in the correlation integral determine the value of ApEn.

An example will clarify the process of calculating ApEn. Consider the following finite series of data *u*:

{−0.5,−0.4,−0.3,−0.2,−0.1,0,0.1,0.2,0.3,0.4,0.5,0.4,0.3,0.2,0.1,0,−0.1,−0.2,−0.3,−0.4,−0.5}

First, it is required to set *m*, the length of the compared blocks, and *r*, the noise filter. The idea of ApEn is to divide the series into blocks and compare their similarity. In this example we use *m* = 2. Starting with the first block x(1)=[u(1),u(2)]=[−0.5,−0.4], we compare the rest of the blocks of length m=2 with x(1) to see if the difference between all the positions of the vectors is less than or equal to *r*. If so, we consider that block “possible”, and we compare then the next position, m+1, the third position in this example.

If the requirement that the distance between the following positions is less than or equal to *r* is met, we consider that the block is a “match”. Once we have done that process with all the blocks, the conditional probability ($C_{i}$) is the ratio between the matches (number of instances that: |u(j)−u(i)|≤r and |u(j+1)−u(i+1)|≤r and … and |u(j+m)−u(i+m)|≤r), and possibles (those cases in which only vectors of length *m* fulfilled the condition of being close to each other, but the difference in the following component m+1 is greater than *r*; formally |u(j)−u(i)|≤r and |u(j+1)−u(i+1)|≤r … |u(j+m−1)−u(i+m−1)|≤r, but |u(j+m)−u(i+m)|≥r).

In the previous example, we set m=2 and r=0.15, and starting with x(i)=x(1)=[u(1),u(2)]=[−0.5,−0.4] as a template we compare it with all the blocks in the sequence. First, it would be compared to itself, being an obvious coincidence (note that this “self-counting” implies a statistical bias that is extremely important in situations with a small amount of data; it will be discussed later when the properties of ApEn are detailed). Then we compare the following block x(j)=x(2)=[u(2),u(3)]=[−0.4,−0.3] with x(1) component to component. The distance between the first components is |d[u(1)−u(2)]|=|d[−0.5,−0.4]|=|−0.1|≤0.15. As the condition meets, we evaluate the second component |d[u(2)−u(3)]|=|d[−0.4,−0.3]|=|−0.1|≤0.15. As the condition for all the components {1,…,m} is met, these two blocks of length *m* are possible, and therefore we evaluate the following component m+1 after those two blocks to see if that component continues meeting the condition, i.e., if there is a pattern. In this example, it would be |d[u(3)−u(4)]|=|d[−0.3,−0.2]|=|−0.1|≤0.15, which in this case would be a match, meaning that the pattern continues the same after the two blocks.

Then we would evaluate the next block, x(3)=[u(3),u(4)]=[−0.3,−0.2] with x(1), and it is easy to prove that it is not a possible block since |d[u(1)−u(3)]|=|d[−0.5,−0.3]|=|−0.2|≥0.15, i.e., it does not meet the condition even for the first component, so it is not necessary to check the rest of the components. As we see, the choice of the noise filter *r* determines this process; larger values of *r* would allow it to be a possible block. In the same way, we would compare the rest of the blocks in the sequence.

**Figure 2:**
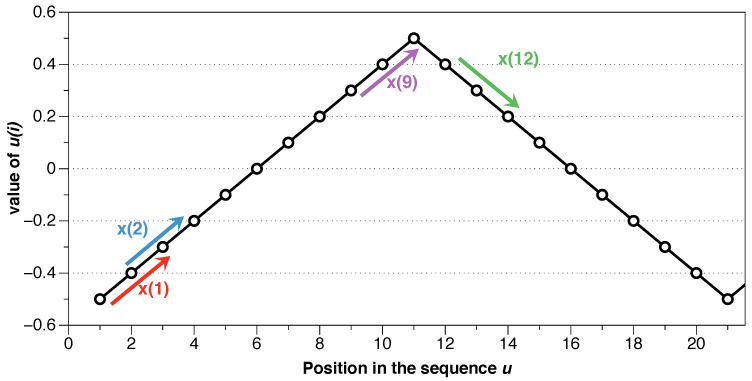
Example for series u={−0.5,…,0,…,0.5,…,0,…,−0.5}.

Once we have compared all the blocks with x(1), we choose x(2) as a template and continue in the same way. Let’s focus, for example, on using the block x(9) as a template. It is easy to prove that the block x(8) is a match. However, let’s evaluate x(12) versus x(9). The first component is fulfilled since |d[u(9)−u(12)]|=|d[0.3,0.4]|=|0.1|≤0.15. The requirement for the second component is also met |d[u(10)−u(13)]|=|d[0.4,0.3]|=|0.1|≤0.15, being therefore possible. However, the next position does not, |d[u(11)−u(14)]|=|d[0.5,0.2]|=|0.3|≥0.15, and, therefore, it is not a match. Seeing Figure 2 it is visually obvious that x(9) does not have the same pattern as x(12), and that is reflected in not being a match. While x(9) follows a pattern of up-up, x(12) follows a down-down pattern.

Once we have evaluated all the blocks, we can determine the conditional probabilities by simply dividing matches/possibles. Those probabilities are the core of the ApEn definition.

For example, if we evaluate the series u={−0.5,−0.4,−0.3,−0.2,−0.1,0,0.1,0.2,0.3,0.4,0.5}, which would be a straight line from −0.5 to 0.5, the probabilities for each block (considering m=2 and r=0.15 as above) are shown in Table 1.

With this table of probabilities, we can calculate the average of the logarithm of those probabilities (matches/possibles), and define ApEn as the negative value of that quantity [20]. In the example shown in Table 1 the probability is one, and the logarithm is zero, being ApEn equal to zero, implying that the series is entirely predictable.

**Figure 3:**
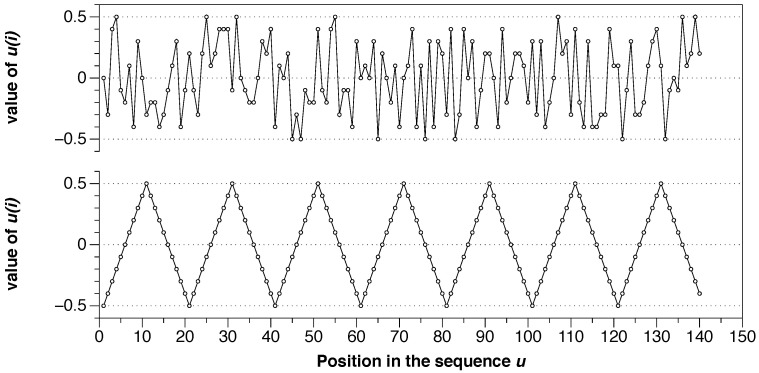
(**top**) Series *u* with 140 shuffled points with approximate entropy (ApEn) = 0.605. (**bottom**) Example for the series of 140 organized points u={−0.5,−0.4,…,−0.4,−0.5} with ApEn = 0.0.

We can extrapolate the previous series of numbers to a broader situation with, for example, 140 points by simply repeating u={−0.5,−0.4,−0.3,−0.2,−0.1,0,0.1,0.2,0.3,0.4,0.5} seven times. The bottom of Figure 3 shows that new series. Also, we can take the same series and shuffle it in a pseudo-random way by using computer software. Here we use the programming language R and the functions “set.seed(001)” and “sample(sample(u))” to shuffle the series, with the result shown at the top of the Figure 3.

These two series have the same moment statistics values (mean, variance, etc.), but their degree of randomness is different. Following the ApEn algorithm, we can calculate the exact number, and we obtain ApEn = 0.00 for the organized series (bottom) and ApEn = 0.605 for the shuffled version (top). It is visually evident that the organization at the bottom is utterly predictable, but the same data at the top have a high level of randomness. ApEn measures it and gives low values to organized series with apparent patterns and higher values to random series.

We present now the properties of ApEn in the form of a list.

- The ApEn statistic is independent of any model, which makes it suitable for the analysis of data series without taking into account anything else but the data.
- ApEn is very stable to large and infrequent outliers or numerical artifacts, like Shannon entropy. Large outliers are low probability events, which make a small contribution to the general measure. These events, however, are critical in moment statistics, since the differences between the values and the mean are quantified. Variability can detect deviations from the average value, but it does not worry about the regularity of the data. The best analysis of a series of data would be the combined use of regularity statistics and moment statistics. In this sense, ApEn has been shown to distinguish normal from abnormal data in instances where moment statistic approaches failed to show significant differences [21].
- Due to the construction of ApEn and its foundations in IT, the value of ApEn is non-negative. It is finite for stochastic processes and for deterministic processes with noise. ApEn has a value of 0 for perfectly regular series (as series A in the Section 2.1 or in the previous example).
- ApEn takes the value ln2 for totally random binary series, and, in general, the value lnk for alphabets of *k* symbols [22].
- ApEp is not altered by translations or by scaling applied uniformly to all terms. ApEn is not invariant under coordinate transformations, hence scale must be fixed. Such non-invariance is also common to the differential entropy [19].
- In the original definition of ApEn, preserving order is a relative property. ApEn can vary significantly with the choice of *m* and *r*, so it is not an absolute measure. The key to ApEn’s utility is that its relativity is enough to discriminate systems. According to Pincus, in general, given two data series *A* and *B*, when ApEn$\left(m_{1},r_{1}\right)\left(A\right)\leq$ ApEn$\left(m_{1},r_{1}\right)\left(B\right)$ then ApEn$\left(m_{2},r_{2}\right)\left(A\right)\leq$ ApEn$\left(m_{2},r_{2}\right)\left(B\right)$. However, in reality, this is not a general characteristic of ApEn, some pairs are fully consistent, but others are not [20].
- Non-linearity causes a greater value of ApEn [20].
- According to Pincus, from the statistical perspective, it is imperative to eliminate any trend before making meaningful interpretations from the statistical calculations [20]. However, the use of ApEn with raw data series has shown its effectiveness; in the next section we will discuss this in more detail.
- Any steady-state measure that emerges from a model of a deterministic dynamic system can be approximated with arbitrary precision by means of a stochastic Markov chain process [20]. ApEn is part of a general development as an entropy rate for a Markov chain which approximates a process [23], hence it can be used in deterministic and stochastic systems.
- Recommended values: *m* must be low, *m* = 2 or 3 are typical options, and *r* must be large enough to have a sufficient number of sequences of *x*-vectors within a distance *r* from most of the specified vectors, to ensure reasonable estimates of conditional probabilities. The recommended *r* values are generally in the range of 0.1 to 0.25 standard deviation of the series of data under analysis. ApEn(m,r) grows with a decrease of *r* as log(2r), exhibiting an infinite variation with *r*, which implies a great variation in the value of the statistic ApEn(m,r,N) with *r*. We will discuss about the selection of the parameter *r* and how it connects with the explained theoretical framework in the next Section.
- The number of data required to discriminate between systems is in the range of $10^{m}$ to $30^{m}$, as in the case of chaos theory, but since *m* is usually a low value, even a series with a small number of data such as *N* = 100 is adequate for the analysis.
- Systems with a signal-to-noise ratio lower than three, i.e., situations in which the noise is substantial, would compromise the validity of ApEn calculations.
- The greater utility of ApEn arises when the means and the standard deviations of the systems show few changes with the evolution of the system. To compare different data series, it is recommended to normalize these series with respect to their standard deviation before the comparison $u^{*}\left(i\right)=\left(u\left(i\right)-mean\left(u\right)\right)/sd\left(u\right)$.
- The ApEn algorithm makes use of the data vector $\left\{x_{1},x_{2},\ldots ,x_{T}\right\}$ instead of using the probabilities associated with the occurrence of each result $\left\{p_{1},p_{2},\ldots ,p_{T}\right\}$. ApEn is directly applicable without knowing or assuming anything about the dataset or knowing anything about the process that generates the values.
- The ApEn algorithm require equally spaced measurements over time [7].
- ApEn is a biased statistic. ApEn(m,r,N) increases asymptotically with *N* to ApEn(m,r), and the bias arises from two separate sources. First, in the calculation of the correlation integral $C_{i}^{m}\left(r\right)$ the vector x(i) counts itself to ensure that the logarithms remain finite, underestimating the calculation of conditional probabilities as a consequence of it. If the number of matches between templates is low, the bias can be as high as 20% or 30%. The higher the number of points *N*, the lower the bias. Second, the concavity of the logarithm implies a bias in all the regularity statistics mentioned in the previous sections. The correlation integral $C_{i}^{m}\left(r\right)$ is estimated from the sample value, but the Jensen inequality implies that E[log(X)]<log(E[X]) [21].
- ApEn exhibits asymptotic normality. The detailed demonstrations can be found in [24,25].
- The standard deviation of ApEn(2, 0.15σ, 1000) determined through Monte Carlo simulations is less than 0.0055 for a large class of models, which indicates that ApEn’s small error bars provide practical utility for the analysis of data.
- If u={u(1),u(2),…,u(N)},N≥1 is a typical realization of a Bernuilli process, then $\lim_{N\to \infty }ApEn\left(m_{crit}\left(N\right),N\right)\left(u_{N}\right)=h=$ entropy of the process [26].

We finish this section by mentioning an aspect of ApEn which is essential to be able to obtain information objectively and comparable to other data sets, which is how to select the value of *r*.

In the previous section we saw that the KS entropy is related to information theory. ApEn is related to the conditional entropy up to the point that if *r* is small enough, ApEn(m,r) = $H(X_{m+1}|X_{1},\ldots ,X_{m})$. The entropy rate $\lim_{n\to \infty }H\left(X_{n}|X_{1},\ldots ,X_{n-1}\right)$ is the discrete state analog of the KS entropy (it is not possible to move from the discrete state to the continuum as a limit. The interested reader should seek information about information theory in continuous states. See for example [8]). For a Markov chain (m,ϵ,A,B) in the interval [A,B] divided into cells of size ϵ, with π as a stationary measure for the Markov chain, almost certainly for a value r<ϵ (Theorem 1 in [23], using the same notation):

(18) $$ApEn\left(m,r\right)=-\sum_{ivect\in \Gamma^{m}}\sum_{j\in \Gamma }\pi \left(ivect\right)p_{ivect,j}\log\left(p_{ivect,j}\right).$$

where the right side is the entropy rate.

Pincus proposed the use of *r* between 0.1 and 0.25 standard deviations of the series because those values are generally useful for slow-dynamics systems. However, although the range may be appropriate for some situations, it is not a clear guide to what precise value we should use, and the results may change depending on the exact number.

The question arises as to what value of *r* should be used for the comparison, and is of critical importance for data series where relative consistency is not guaranteed since it can lead to an incorrect evaluation of the complexity of a series of data. Two series are completely consistent if ApEn$\left(m_{1},r_{1}\right)\left(A\right)\leq$ ApEn$\left(m_{1},r_{1}\right)\left(B\right)$ then ApEn$\left(m_{2},r_{2}\right)\left(A\right)\leq$ ApEn$\left(m_{2},r_{2}\right)\left(B\right)$ for any $r_{2}\geq r_{1}$.

The recommended value of *r* may be useful for slow dynamic series such as heart rate or hormone release but is not always appropriate for fast dynamics such as neural signals or the stock market. Previous research has suggested that the use of the value of *r* which maximizes ApEn (MaxApEn) quantifies the greatest difference in information between the segments of length *m* and m+1, and therefore the selection of $r_{max}$ allows for greater signal complexity than other values of *r* [27]. Simulations and research with real clinical data have validated the results [28], using that election with a “heuristic” justification. It should be noticed that the value of *r* for which the maximum value of ApEn is reached increases as the length of the data decreases, and therefore it may not be within the recommended range. An automatic method has recently been proposed to determine the value of $r_{max}$ [29].

As stated before, it was only after the publication of Shannon’s purely mathematical work when researchers established a connection between IT and physics. This interconnection has proven to be valuable for both physics and information theory. In physics, entropy is a measure of the disorder of a system, and without being very rigorous it can be said that the highest level of entropy determines the equilibrium of the system, i.e., it follows a situation of maximum entropy. Imagine a room filled with two gases separated by a barrier. If we eliminate that barrier, the gases will mix until obtaining a perfectly homogeneous situation, an entirely disordered system where the gas is distributed equally in each location of the room, having the system a maximum entropy. A hypothetical situation where each gas would go to a different corner of the room is extremely unlikely, i.e., the probability of that event is very concentrated and, therefore, the uncertainty is low, as well as the entropy (Figure 1).

The roots of ApEn are in information theory, being ApEn an entropy rate. By selecting MaxApEn, we would be selecting the value of *r* which maximizes that rate, and the reader can remember the discussion of Jaynes’ maximum entropy principle. The maximum entropy principle is an efficient information processing rule, with the total input information equal to the total output information. MEP uses all available information without making another assumption, and it is presented as the best approach to determine probabilities. Equation (18) shows the probabilities of the entropy function, and their maximization under the MEP principle implies maximization of ApEn. While this is far from being a theoretical demonstration, it does provide an insight into why it is said that MaxApEn best serves the purpose of correctly determining the complexity of the system. In the next chapter, we will see that it is essential to select a value of *r* equal or greater than the point of maximum ApEn.

### 3.2. Cross-ApEn

It is possible to study the degree of correlation of two series by analyzing the patterns of one series in the data from another. This idea is known as cross-ApEn, a measure of the asynchrony between two different time series.

Conceptually, the formulation is very similar to ApEn, with the peculiarity that now we compare the blocks of a series with the blocks of the other series, instead of doing it with the same series. A low number of coincidences imply a high value of cross-ApEn, indicating asynchrony. If the value of cross-ApEn is low, then the two series are more concordant. The idea behind cross-ApEn is the same as before; it is not necessary to model the system in order to discriminate it satisfactorily.

Formally, let u={u(1),u(2),…,u(N)} and v={v(1),v(2),…,v(N)} be two series of length *N*. Given a value *m*, we define the blocks x∈u and y∈v as x(i)=(u(i),u(i+1),…,u(i+m−1)) and y(i)=(v(i),v(i+1),…,v(i+m−1)). For each i≤N−m+1 we calculate the correlation integral as $C_{i}^{m}\left(v||u\right)$ = (number of j≤N−m−1 for which d[x(i),y(j)]≤r)/(N−m−1), where $d\left[x\left(i\right),y\left(j\right)\right]=max_{k=1,2,\ldots ,m}(|u\left(i+k-1\right)-v\left(j+k-1\right)|)$ is the maximum distance of its scalar components. In this algorithm, $C_{i}^{m}\left(r\right)$ measures for each *i* the regularity or frequency of patterns in the series *v* similar to a given pattern from the series *u* of length *m* taken as a template, with a tolerance *r*.

Once the correlation integral is calculated, the following is defined:

(19) $$\phi^{m}\left(r\right)\left(v||u\right)=\frac{1}{N-m+1}\sum_{i=1}^{N-m+1}\log C_{i}^{m}\left(v||u\right),$$

and cross-ApEn is calculated as:

(20) $$cross-ApEn\left(m,r,N\right)\left(v||u\right)=\phi^{m}\left(r\right)\left(v||u\right)-\phi^{m+1}\left(r\right)\left(v||u\right).$$

The idea of building cross-ApEn is to compare sequences of processes in a network with related variables [26]. The use of this correlation measure in clinical research showed that in situations in which there are no differences in mean values or apparent correlation (low Pearson’s R), it is possible to differentiate groups and find a correlation based on pattern analysis. ApEn and cross-ApEn are especially useful for the characterization of non-linear systems, where other techniques are less effective when determining characteristics of the models [30].

As in the previous section, we show below the properties of cross-ApEn in the form of a list:

- cross-ApEn is used with stationary series σ=1.
- As the cross-ApEn algorithm analyzes one series versus another, there is no self-counting, so that source of bias which was present in ApEn does not exist in cross-ApEn. However, since the definition is equally logarithmic, it is required that there is at least one match in the pattern count in order to avoid the calculation of a logarithm of zero, situation in which cross-ApEn would remain undefined.
- Like ApEn, it does not verify relative consistency for all data series; therefore, the synchrony or asynchrony between series may depend on the chosen parameters.
- The use of two series, one as template and another as target, makes the analysis directional and depends on which one is the template series and which one is the target one, i.e., in general, cross-ApEn(m,r,N)(v||u)≠ cross-ApEn(m,r,N)(u||v). If we remember the section on information theory, the Kullback-Leibler distance had the same limitation, D(p||q)≠D(q||p).
- cross-ApEn may fail to judge the synchrony order of two series with respect to a third one [31], situation derived from the lack of relative consistency.

Even though a wide variety of investigations have made use of ApEn, the use of cross-ApEn has not been so widespread since the results are not always consistent. The fundamental reason for this inconsistency is the low fidelity of the estimation of conditional probabilities in some situations. However, it is possible to define a set of parameters to characterize the stability regions mathematically [32].

The existence of bias in ApEn has given rise to the proposal of other statistics of regularity to try to avoid self-counting. The next section explains sample entropy (SampEn), a statistics proposed as an alternative to ApEn.

### 3.3. Sample Entropy

In practice, the ApEn bias has two important implications. The first one is that, as mentioned previously, the relative consistency is not guaranteed, and depending on the value of *r* the results may be different. The second one is that the value of ApEn depends on the length of the data series. To avoid those two problems, Richman and Moorman [31] defined SampEn, a statistic which does not have self-counting. SampEn(m,r,N) is the negative value of the logarithm of the conditional probability that two similar sequences of *m* points remain similar at the next point m+1, counting each vector over all the other vectors except on itself. It implies that SampEn maintains the relative consistency and is also mostly independent of the length of the series.

According to [31], the bias of ApEn makes the results suggest more regularity than there is in reality. By allowing each vector to count itself, all functions $C_{i}^{m}\left(r\right)$ remain positive, avoiding a situation in which we would have to calculate a log(0). The functions $C_{i}^{m}\left(r\right)$ are, basically, conditional probabilities calculated as a sum of the (matches)/(total of possible vectors) among all the target vectors, and the self-counting allows at least one match and one possible vector. If we call $B_{i}$ to the possible vectors and $A_{i}$ to all the matches, in reality, the ApEn algorithm calculates ($A_{i}$ + 1)/($B_{i}$ +1), which is always greater than the amount without bias which should be measured, ($A_{i}$)/($B_{i}$). This bias is obviously more important for samples with a small number of points *N*. Eliminating this bias by preventing each vector from being counted with itself would make ApEn unstable in many situations, leaving it undetermined if each vector does not find at least one match.

SampEn solves the self-counting problem to eliminate that bias. According to its authors, eliminating self-counting is justified given that “entropy is conceived as a measure of the rate of information production, and in this context comparing data with themselves is meaningless” [31]. SampEn does not use the same pattern approach than ApEn to determine its value, using instead the whole series together, requiring only that a template vector find a match of length m+1 to be defined. It contrasts with ApEn, where each template vector has to find a match to be defined.

The SampEn algorithm is as follows. We define the total number of possible vectors by calculating for each template vector:

(21) $$\begin{array}{ccc} B_{i}^{m}\left(r\right) & =\frac{1}{N-m-1}\times & [\mathrm{number}\;\mathrm{of}\;\mathrm{vectors}\;x_{m}\left(j\right)\;\mathrm{at}\;a\;\mathrm{distance}\;r\;\mathrm{of}\;x_{m}\left(i\right),\\ & & \;\mathrm{without}\;\mathrm{allowing}\;\mathrm{self}-\mathrm{counting},\;\mathrm{where}\;j=1,N-m]\\ & =\frac{1}{N-m-1} & \sum_{j=1,j\neq i}^{N-m}\left[\mathrm{number}\;\mathrm{of}\;\mathrm{times}\;\mathrm{that}\;d[\right|x_{m}\left(j\right)-x_{m}\left(i\right)|]<r] \end{array}$$

and adding all the template vectors:

(22) $$\begin{array}{cc} B^{m}\left(r\right) & =\frac{1}{N-m}\sum_{i=1}^{N-m}B_{i}^{m}\left(r\right)\\ & =\frac{1}{N-m-1}\frac{1}{N-m}\sum_{i=1}^{N-m}\sum_{j=1,j\neq i}^{N-m}\left[\mathrm{number}\;\mathrm{of}\;\mathrm{times}\;\mathrm{that}\;d[\right|x_{m}\left(j\right)-x_{m}\left(i\right)|]<r] \end{array}$$

In the same way, we define the total number of matches by calculating for each model vector:

(23) $$\begin{array}{ccc} A_{i}^{m}\left(r\right) & =\frac{1}{N-m-1}\times & [\mathrm{number}\;\mathrm{of}\;\mathrm{vectors}\;x_{m+1}\left(j\right)\;\mathrm{at}\;a\;\mathrm{distance}\;r\;\mathrm{of}\;x_{m+1}\left(i\right),\\ & & \;\mathrm{without}\;\mathrm{allowing}\;\mathrm{self}-\mathrm{counting},\;\mathrm{where}\;j=1,N-m]\\ & =\frac{1}{N-m-1} & \sum_{j=1,j\neq i}^{N-m}\left[\mathrm{number}\;\mathrm{of}\;\mathrm{times}\;\mathrm{that}\;d[\right|x_{m+1}\left(j\right)-x_{m+1}\left(i\right)|]<r] \end{array}$$

and adding them as:

(24) $$\begin{array}{cc} A^{m}\left(r\right) & =\frac{1}{N-m}\sum_{i=1}^{N-m}A_{i}^{m}\left(r\right)\\ & =\frac{1}{N-m-1}\frac{1}{N-m}\sum_{i=1}^{N-m}\sum_{j=1,j\neq i}^{N-m}\left[\mathrm{number}\;\mathrm{of}\;\mathrm{times}\;\mathrm{that}\;d[\right|x_{m+1}\left(j\right)-x_{m+1}\left(i\right)|]<r] \end{array}$$

Therefore, $B^{m}\left(r\right)$ is the probability that two sequences are similar for *m* points (possibles), while $A^{m}\left(r\right)$ is the probability that two sequences are similar for m+1 points (matches). Since the number of matches is always less than or equal to the number of possible vectors, the ratio $A^{m}\left(r\right)/B^{m}\left(r\right)$ is a conditional probability less than unity.

The parameter Sample Entropy is defined as SampEn(m,r) = $\lim_{N\to \infty }\left\{-\log\left[A^{m}\left(r\right)/B^{m}\left(r\right)\right]\right\}$, value which is estimated from the statistic SampEn(m,r,N) = $-\log[A^{m}\left(r\right)/B^{m}\left(r\right)]$.

It is essential at this point to explain the differences regarding the theoretical formulation between ApEn and SampEn, remembering the formalism presented in the Section 2.3.

Richman and Moorman defined the SampEn statistic to analyze the randomness of a series through the direct use of the correlation integrals defined by Grassberger and collaborators (Equation (12), using Takens’ metric in Equation (13)). Pincus, however, does not make a direct use of the correlation integrals, but instead bases Approximate Entropy on the work of Eckmann and Ruelle, defining the functions ϕ (Equation (14)) and subsequently calculating the entropy as a difference of those functions (Equation (15)).

Mathematically, the differences are the following. The calculation of possible and matching vectors for SampEn has just been detailed in Equations (22) and (24). For ApEn, we can algebraically manipulate its definition to get [31]:

(25) $$ApEn\left(m,r,N\right)=\frac{1}{N-m-1}\sum_{i=1}^{N-m+1}\log\left[C_{i}^{m}\left(r\right)\right]-\frac{1}{N-m}\sum_{i=1}^{N-m}\log\left[C_{i}^{m+1}\left(r\right)\right].$$

When *N* is large, ApEn can be approximated by:

(26) $$ApEn\left(m,r,N\right)≃-\frac{1}{N-m}\sum_{i=1}^{N-m}\log\left[C_{i}^{m+1}\left(r\right)\right]=-\frac{1}{N-m}\sum_{i=1}^{N-m}\log\left[A_{i}/B_{i}\right].$$

The error committed in this approximation is <0.05 for N−m+1>90 and <0.02 for N−m+1>283 [31]. This approximation is used in the FORTRAN algorithm provided by Pincus [33] (although he does not mention it), and it is the same algorithm that is used in this work and presented in Appendix A. In this way we can compare the equations to calculate both statistics:

(27) $$\begin{array}{c} SampEn\left(m,r,N\right)=-\log\frac{\sum_{i=1}^{N-m}\sum_{j=1,j\neq i}^{N-m}\left[\mathrm{number}\;\mathrm{of}\;\mathrm{times}\;\mathrm{that}\;d[\right|x_{m+1}\left(j\right)-x_{m+1}\left(i\right)|]<r]}{\sum_{i=1}^{N-m}\sum_{j=1,j\neq i}^{N-m}\left[\mathrm{number}\;\mathrm{of}\;\mathrm{times}\;\mathrm{that}\;d[\right|x_{m}\left(j\right)-x_{m}\left(i\right)|]<r]}\\ ApEn\left(m,r,N\right)≃-\frac{1}{N-m}\sum_{i=1}^{N-m}\log\frac{\sum_{j=1}^{N-m}\left[\mathrm{number}\;\mathrm{of}\;\mathrm{times}\;\mathrm{that}\;d[\right|x_{m+1}\left(j\right)-x_{m+1}\left(i\right)|]<r]}{\sum_{j=1}^{N-m}\left[\mathrm{number}\;\mathrm{of}\;\mathrm{times}\;\mathrm{that}\;d[\right|x_{m}\left(j\right)-x_{m}\left(i\right)|]<r]}. \end{array}$$

Thus, we can see that the differences between SampEn and ApEn are essentially three:

- SampEn does not allow self-counting (j≠i) while ApEn does.
- The sum of all template vectors is inside the logarithm in SampEn and outside in ApEn. It implies that SampEn considers the complete series and if a template finds a match, then SampEn is already defined, while ApEn needs a match for each template. The Jensen’s inequality tells us that log(∑)>∑log, so that term is greater in SampEn than in ApEn.
- ApEn includes a factor $\frac{1}{N-m}$, which makes this statistic dependent on the size of the series, whereas SampEn does not include it.

A priori, it seems that the use of SampEn eliminates many of the problems associated with ApEn, being, according to its authors, useful to quantify regularity in a system more effectively. However, a theoretical clarification has to be done to understand the implications of using one algorithm or another.

In Section 2.2 we presented the theoretical framework of information theory, and in Section 2.3 we used that knowledge to explain how to quantify the regularity of a system. Shannon entropy was the base of all the measurements in that section, and in particular, the entropy measure of Eckmann and Ruelle presented in the Equation 15 is an exact measure of the entropy of the system [18] (p. 650), an average rate of information creation.

However, the use of the correlation integrals directly allows us to obtain an estimate of Rényi entropy [16]. Without going into much detail, this entropy is a generalization of the entropy function that tends to Shannon’s function when a specific parameter α tends to 1. When the parameter α tends to zero, Rényi entropy assesses all possible events more equitably, regardless of their probabilities. The higher the value of α, the more important the unlikely events are, contrary to what happened with Shannon entropy where low probability events did not contribute much.

The use of the correlation integrals in SampEn, motivated by the works of Grassberger and his coauthors, gives an estimate of Rényi entropy of order 2. One property of Rényi entropy is that $K_{q}>K_{q^{\prime }}$ for all $q^{\prime }>q$, where *K* means entropy. Therefore $K_{1}>K_{2}$, being the estimates which make use of the correlation integrals a lower bound to the real value of the Kolmogorov–Sinai entropy.

Furthermore, for Rényi entropy of order two to be a correct estimate, the dimension needs to tend to infinity (or, at least, be sufficiently high). Low values of the dimension may overestimate the real value of the KS entropy, as can be seen in the Henon map of Figure 4 in [16]. Also, higher values of the dimension can “seriously underestimate the value of entropy” (mean rate of information creation) [34].

### 3.4. Cross-SampEn

To finish this chapter, we define cross-SampEn following the same ideas that motivated the definition of cross-ApEn. The most remarkable feature of this statistic is its independence of directionality, i.e., it does not matter which series is the template and which one is the target since the results are the same, cross-SampEn(m,r,N)(v||u) = cross-SampEn(m,r,N)(u||v).

Similar to SampEn, we define $B_{i}^{m}\left(r\right)\left(v||u\right)$ as ${\left(N-m\right)}^{-1}$ number of times that the vector $y_{m}\left(j\right)$ is at a distance less than *r* of the vector $x_{m}\left(i\right)$, where *j* goes from 1 to N−m. Adding all the values in *i*, we define $B^{m}\left(r\right)\left(v||u\right)={\left(N-m\right)}^{-1}\sum_{i=1}^{N-m}B_{i}^{m}\left(r\right)\left(v||u\right)$. On the other hand, we define $A_{i}^{m}\left(r\right)\left(v||u\right)$ as ${\left(N-m\right)}^{-1}$ number of times that the vector $y_{m+1}\left(j\right)$ is at a distance less than *r* of the vector $x_{m+1}\left(i\right)$, where *j* goes from 1 to N−m.

Cross-SampEn is defined as cross-SampEn$\left(m,r,N\right)\left(v||u\right)=-\ln\{[A^{m}\left(r\right)(v||u)]/[B^{m}\left(r\right)(v||u)]\}$. As the authors indicate, by examining these definitions we find that $\left(N-m\right)B_{i}^{m}\left(r\right)\left(v||u\right)$ is the number of *v* vectors which are at a distance less than *r* from the template *i*-th from the *u* series. Summing over all the templates, we see that $\sum_{i=1}^{N-m}\left(N-m\right)B_{i}^{m}\left(r\right)\left(v||u\right)$ simply counts the number of vectors of the two series that match at a distance *r*. This number of vectors is independent of which one is the template series and which one is the target series, implying that cross-SampEn is independent of the direction, i.e., cross-SampEn(m,r,N)(v||u) = cross-SampEn(m,r,N)(u||v).

## 4. ApEn and SampEn: Step by Step Tutorial

In this section we provide a detailed set of instructions to calculate the approximate entropy and sample entropy of a dataset. While the previous sections where dedicated to understand the theory behind the algorithms, we have written this section in a way that any researcher could understand how to use the algorithms without knowing anything about information theory or chaos theory. That configuration is useful because this tutorial is intended to provide an understanding of the algorithms for researchers in different fields, and therefore we pay more attention to the process than to the analysis of the results.

### 4.1. Approximate Entrpy of the New York City Temperature

The ApEn algorithm has been explained theoretically in many publications by Pincus and coauthors. In this paper we explain the algorithm following the steps in [33] and illustrating it with an example. We recall that the negative value of ApEn is calculated as:

–ApEn = $\phi^{m+1}\left(r\right)-\phi^{m}\left(r\right)$ = average over *i* of ln [conditional probability that |u(i+m)−u(j+m)|≤r, given the fact that the previous values fulfill the condition |u(i+k)−u(j+k)|≤r for k=0,1,…,m−1]

STEP 1: to calculate ApEn we need a series of data with the same temporal difference between them; let us call this series *u*. In our example, we will use the local temperature of a location in New York City (Zip code 10023) since 1869. The data can be freely downloaded from the NOAA National Centers for Environmental Information (www.ncdc.noaa.gov). Lines 1, 2 in Appendix A load the data series into R.

STEP 2: set the ApEn parameters, namely the embedding dimension *m* and the noise filter *r*. In our code example, we use m=2 and r=0.2σ in lines 4–12. Also, it is recommended to make the data series stationary by subtracting the mean and dividing it by the standard deviation. ApEn could be applied to the raw data, as we do in the next steps, but always being careful with the interpretation.

STEP 3: create the sequence of all possible vectors of length *m*. Each of those vectors will be used as a template once, and will serve for the comparison with all other vectors in the sequence. In Appendix A, we select the template of length *m* (line 23) and compare it with all possible vectors of length *m* in the data series (line 28).

STEP 4: compare every vector of longitude *m* with all the possible vectors of the same length within the noise filter level. We start by creating a vector x(i)=[u(i),…,u(i+m−1)]. In our data set of length *N*, the first values of the temperature data series (in ${}^{{}^{\circ}}$F) are u={27,35,37,43,38,…}. If we set m=2, then our first template vector would be x(i)=x(1)=[27,35], and we would compare it with all other possible vectors x(j) for j=1,…,N−m to see if d[x(i),x(j)]=max|u(i)−u(j)|≤r in a component wise way. If it fulfills the condition it would be a possible vector, and then we would compare the next position m+1 to see if it is a match. First, we would compare x(1) with x(1), then x(1) with x(2), … Once we have compared x(1) with all the possible vectors, we would set x(2) as template and do the same process.

**Figure 4:**
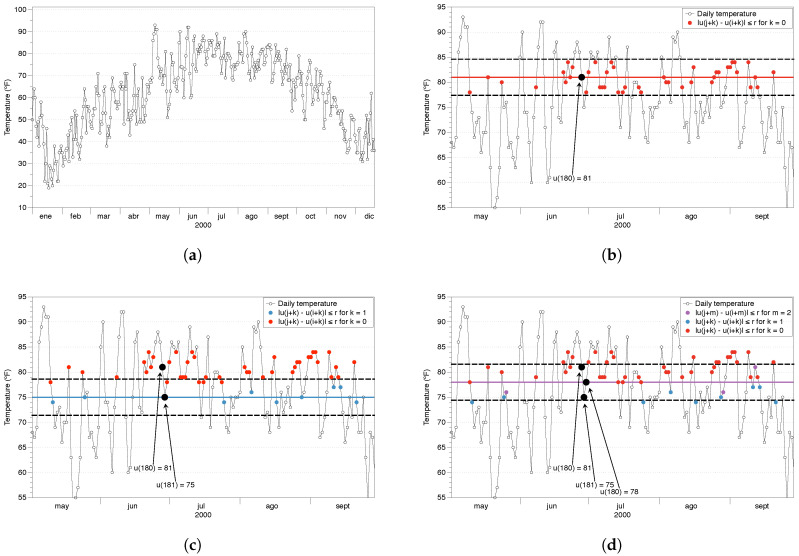
NYC temperatures and ApEn parameters r=0.2σ and m=2. (**a**) Neq York City (NYC) temperature for the year 2000. (**b**) Red points verify that d[x(i+k),x(j+k)]≤r for k=0. (**c**) Blue points verify that d[x(i+k),x(j+k)]≤r for k=1. (**d**) Purple points verify that d[x(i+m),x(j+m)]≤r for m=2.

As this is the fundamental process for calculating ApEn, we illustrate it with an example. We have used the temperature of the NYC dataset from 1 January 2000 to 31 December 2001, with a total of N=731 points. In Figure 4a we show the behavior for one year of data, with a clear deterministic pattern (concave function) and stochastic variations every day. The average temperature for that period was 62.13 ${}^{{}^{\circ}}$F and the standard deviation $\sigma =17.55{\;}^{{}^{\circ}}$F. We set the parameters m=2 and r=0.2σ≃$3.5{\;}^{{}^{\circ}}$F. Then, we would chose x(1) as template and compare it with all the vectors of length two in the dataset. Once we are done, we take x(2) and do the same process and so on.

Let us focus for example on using x(180)=[81,75] as template vector. In Figure 4b we show a few points of the series to clearly illustrate the process. To begin with, we check the condition for the first component of value 81 ${}^{{}^{\circ}}$F with all the possible values in the dataset. As the noise filter is $r=3.5{\;}^{{}^{\circ}}$F, we look for those points between 77.4 ${}^{{}^{\circ}}$F and 84.6 ${}^{{}^{\circ}}$F, i.e., we are checking the condition d[x(180+k),x(j+k)]=d[x(180),x(j)]≤r for k=0 (first component) and *j* from 1 to N−m, being *N* the length of the dataset. To illustrate that condition, Figure 4b shows in red color those points that fulfill that condition for a few months.

After checking the first component of the template vector, we check the second component, but only among those vectors which fulfilled the first component, i.e., the red points. Now we check d[x(180+k),x(j+k)]≤r for k=1 (second component), i.e., we check if d[x(181),x(j+1)]≤r for those *j* identified as red points. In our dataset, u(181) = 75 ${}^{{}^{\circ}}$F, and therefore the range of temperatures determined by the noise filter goes now from 71.4 ${}^{{}^{\circ}}$F to 78.6 ${}^{{}^{\circ}}$F. In Figure 4c we identify the points which are in that range in blue color.

The vectors constructed as a red point followed by a blue point are the possible vectors; all those vectors are similar in pattern to the vector x(180)=[u(180),u(181)]=[81,75]. We can see that a different value of the noise filter would change the number of possible vectors. Also, a different value of *m* would imply to check more or less coordinates of the vectors before knowing if those are possible vectors. In the portion of the dataset shown in the figure, we identify 10 possible vectors: nine of those are red-blue vectors and one is the vector used as template (black). This is important because that is a source of bias for ApEn: the self-counting of the template vector.

Once we have evaluated the vectors of length *m* (in our case m=2), we check if the next coordinate follows the same pattern. Formally, we check if d[x(180+m),x(j+m)]=d[x(182),x(j+m)]≤r for m=2 (third component) and those *j* identified as blue points. This time, the value u(182) = 78 ${}^{{}^{\circ}}$F and our noise filter r=0.2σ defines the range from 74.4 ${}^{{}^{\circ}}$F to 81.6 ${}^{{}^{\circ}}$F. We look for those coordinates after a blue point which are in that temperature range, and in Figure 4d we identify them in purple color. In the section of the dataset shown in the picture, only four points fulfill the condition, including the self-counting; those points would be the matches.

We can see that all those four vectors (red-blue-purple and the template vector) have the same pattern, moving down and then moving up, doing a “V” shape. That is the reason why it is said that ApEn is based on counting the patterns within the series of data. Once we have checked x(180) with all the possible vectors of length *m*, we would take the next vector x(181) as template and do the same thing.

In the code presented in Appendix A, this process is carried out in lines 30–63. We start by checking component wise the condition of the noise filter for the different coordinates. Note that in the code, the range of *k* is k=1,…,m+1, but the indexes of the distance functions are calculated as d[x(i+k−1),x(j+k−1)], i.e., it is the same range than before. We have used that notation because we wanted to resemble as much as possible the original code presented in [33].

We check the different possibilities: If d[x(i+k−1),x(j+k−1)]≥r for k<m then we do not have to continue checking the other components because it does not have the same pattern (lines 32–38). If d[x(i+k−1),x(j+k−1)]≤r for k=m, the two vectors are similar and possible (lines 40–49). Then, we check the next position to verify if the pattern continues the same after the two vectors, and if d[x(i+k−1),x(j+k−1)]≤r for k=m+1 then we call those vectors a match (lines 51–60).

STEP 5: once all the vectors have been checked, then we have to determine the ϕ functions defined as:

(28) $$\phi^{m}\left(r\right)={\left(N-m+1\right)}^{-1}\sum_{i=1}^{N-m+1}\ln C_{i}^{m}\left(r\right)={\left(N-m+1\right)}^{-1}\sum_{i=1}^{N-m+1}\ln\frac{matches[i]}{possibles[i]}.$$

We can see that the ratio of matches[i]/possibles[i] is a conditional probability between 0 and 1, which is the core of the algorithm. If the dataset has a strong regularity, then the patterns will be repeated very often and the ratio of matches[i]/possibles[i] will be close to one. The logarithm of those values will be close to zero, implying very low ApEn values. On the other hand, situations with a low number of patterns will have lower conditional probabilities, making the logarithm larger and increasing the value of ApEn. Therefore, low ApEn values indicate the existence of patterns while large values of ApEn indicate more randomness. In Appendix A we calculate the sum in lines 72–75.

STEP 6: as we saw in Equation (26), when *N* is large ApEn can be approximated to ApEn(m,r,N) $≃-\frac{1}{N-m+1}\sum_{i=1}^{N-m+1}\log\left[matches_{i}/possibles_{i}\right]$. In our code, ApEn is simply calculated in line 79 using the previous values of the conditional probabilities.

**Figure 5:**
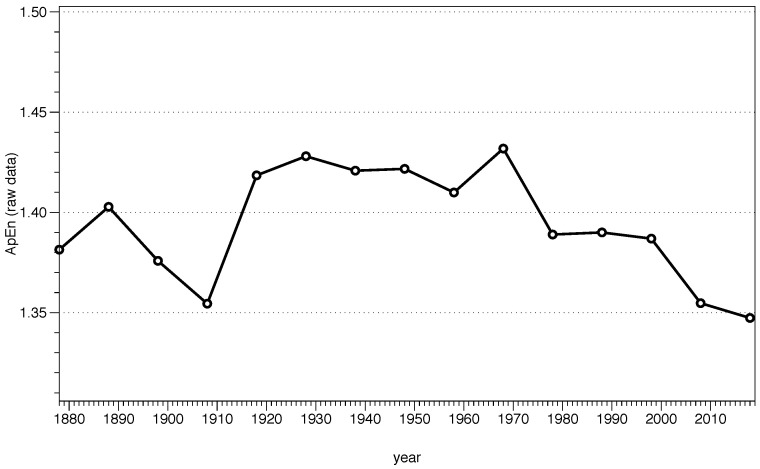
ApEn for *m* = 2 and *r* = 0.2σ of the raw data series every ten years.

We can calculate ApEn following these steps. In Figure 5 we show the results for *m* = 2 and *r* = 0.2σ for sequences of ten years of data starting in 1869. It is important to remark that we are using the raw data, and therefore the interpretation must be done carefully. Let us clarify the situation with an example. Suppose we are analyzing the ApEn of the decade from 1998 to 2008, whose standard deviation is equal to σ = 17.96 ${}^{{}^{\circ}}$F, and our noise filter is *r* = 0.2σ≃ 3.59 ${}^{{}^{\circ}}$F. Looking at Figure 4a, a value of 20 ${}^{{}^{\circ}}$F from January would be compared with data within the range [16.41, 23.59]. It is clear from that figure that we would not be comparing those values with any other moment of the year, since all the temperatures are higher than our range, and in a decadal study we would only be comparing data from January during different years. However, when the block we are analyzing has a value like 55 ${}^{{}^{\circ}}$F, we realize that we can find that value during spring and during fall, finding several possibles and matches vectors. Therefore, the cyclic behavior of the temperature during the year is influencing the results we obtain, and the usefulness of ApEn to determine the complexity could be compromised; if one is using a raw data series, there must be a good reason for it and make a careful interpretation of the results.

Following Pincus, “*If the time series is nonstationary, that is, contains one or more pronounced trends upward or downward, little can be inferred from moment (mean, variability), ApEn, or power spectral calculations, because the trends tend to dominate all other features. [...] From the statistical perspective, it is imperative that any trends be removed before meaningful interpretation can be made from the statistical calculations*” [20]. While ApEn has been used in raw series, it is important to understand that the comparison must be done with homogeneous series, and it is not possible to mix heterogeneous epochs; if the process generating the data changes drastically in different epochs, the alphabet (the possible symbols) change and the value of ApEn changes. Both ApEn and SampEn are relative values which can only be used to compare situations with the same alphabet.

From now on, in this paper we use a stationary version of the data, obtained by taking logarithms to stabilize the variance and then subtracting the previous value of the log to eliminate the trend, $\log(x_{t}/x_{t-1})$. Other ways to normalize the data, such as (u-mean(u))/sd(u), can also be used, and it is fundamental to specify the transformation of the series used in the research.

**Figure 6:**
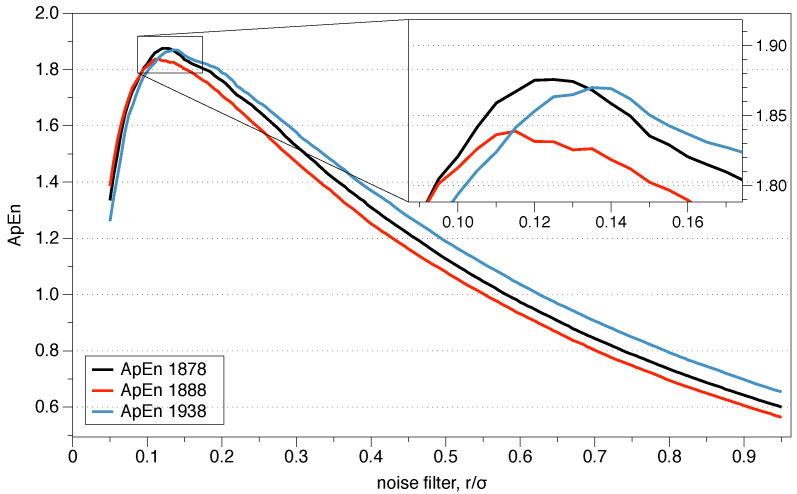
Dependence on *r* of the value of ApEn.

As we have mentioned before, different values of *r* will allow counting different number of patterns, which ultimately determines the value of ApEn. We can analyze the behavior of ApEn depending on the selection of the noise filter. For example, using *m* = 2 and three different sequences of ten years of NYC temperature data: (A) from 1 January 1869 to 31 December 1878, (B) from 1 January 1879 to 31 December 1888, and (C) from 1 January 1929 to 31 December 1938, we obtain the values presented in Figure 6. We recall that the “recommended” range for the *r* is usually between 0.1 and 0.25σ. As we can see in the figure, in this case the maxima for the three sequences of data are within the range. Suppose that we are trying to analyze the randomness of those sequences using ApEn. If we chose *r* = 0.08σ, then the order of the randomness would be 1888 > 1878 > 1938. A value of *r* = 0.10σ would give us 1878 > 1888 > 1938, while for a value of *r* = 0.12σ we would have 1878 > 1938 > 1888. Finally, a value of (or greater than) *r* = 0.15σ would give us 1938 > 1878 > 1888.

As we can see, depending on the selection of the noise filter *r* we can obtain a variety of different results, leading us to different conclusions. As explained in Section 3.1, some researchers have argued that the most reasonable choice is selecting the maximum of the ApEn distribution [27]. In Section 4.3 we will explain why is important to chose a value of *r* greater or equal than the maximum, and keep in mind that the location of the maximum changes with the number of data considered in the analysis and the complexity of the data.

**Figure 7:**
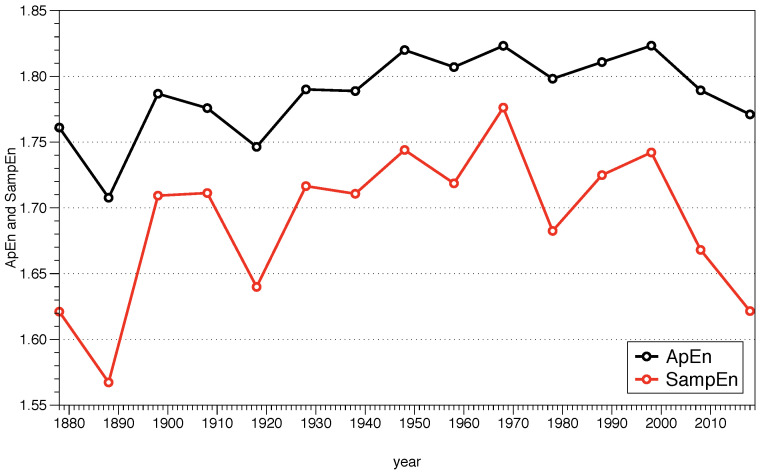
ApEn and SampEn for sequences of ten years of data using *m* = 2 and *r* = 0.2σ.

Using a value of r=0.2σ and m=2, we analyze the Approximate Entropy of the NYC temperatures every ten years, starting with the decade of 1869–1878. Figure 7 shows the results of the analysis. Rather than the scientific implications that it may have, this tutorial is oriented to serve as a guidelines of how to work with ApEn. Therefore, the reader should remember that comparisons are intended between similar processes and that the values are relative. It would not be possible, for example, to compare these values of ApEn with a different process, say the temperature of the ocean, and claim that one is more random than the other just because the values of ApEn are higher. Mathematically, by doing so we would be assuming that those processes have the same alphabet, while there is no reason to claim that. Similarly, it could not be possible to compare our results with the temperatures of the same location in the last million years for example, since the dynamic processes generating those temperatures have changed. In that case it would be key to make the series stationary before applying the algorithm, otherwise the comparison would be pointless.

It is also important to understand that ApEn is an algorithm to differentiate between groups, but it does not give any information about the causes. In that sense, it would be fair to say that according to Figure 7 the randomness of the NYC temperatures analyzed with ApEn seems to be increasing, but it would not be scientifically correct to claim that this is a proof of any kind of climate change. The changes occurred in the city of New York in the last 150 years, such as the buildings, number of people, heating and cooling systems, etc. have had a great importance in the local temperature. Originally, ApEn was developed to classify systems in the medical field, such as heart beats. If two series of heart beat data have different ApEn, then one could infer that the heart has changed, not the reasons why that happened. Certainly a researcher can make any hypothesis, but that is beyond the scope of the use of this algorithm.

### 4.2. Sample Entrpy of the New York City Temperature

The theoretical differences between ApEn and SampEn were outlined in Section 3.3. As explained before, SampEn is an alternative statistic to measure the randomness of a series, defined with the intention of correcting the problems of bias and lack of relative consistency. In addition to these two problems, one of the main arguments mentioned in favor of SampEn is that ApEn gives the data series a degree of regularity higher than the real value, while according to its authors SampEn is a more accurate measure.

In Appendix B we present the corresponding source code in R. The first three steps in the algorithm are the same than before, detailed in lines 1–26. The differences begin in STEP 4, where now it is important to remember that SampEn does not allow self-counting. This condition is easily included in the code in line 30.

STEP 5 differs from ApEn in the way that we determine the probabilities. We recall from Equation (27) that the main difference between the two statistics was how to count the probabilities, which ultimately was determined by the summation over the whole series. Basically, SampEn considers the complete series and if a template finds a match, then SampEn is already defined, while ApEn needs a match for each template. That summation is carried out in lines 73–81.

STEP 6 calculates SampEn simply as the negative logarithm of those probabilities, making a direct use of the correlation integral proposed by Grassberger and coauthors. Lines 83–87 describe the calculation.

**Figure 8:**
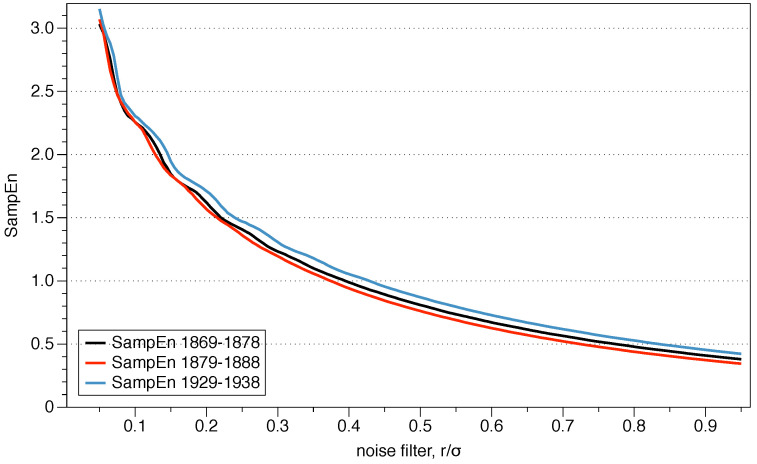
Dependence on *r* of the value of sample entropy (SampEn).

We can evaluate now the dependence on *r* of SampEn. We use the same sequences than we used in Figure 6, and similarly we use *m* = 2. As we can see in Figure 8, SampEn behaves different than ApEn on *r*. While ApEn shows a maximum, SampEn is monotonically decreasing when *r* increases. This monotonous behavior is what we would expect when we analyze the randomness of a series. Also, we can observe that there is no crossing of lines when using SampEn, i.e., the sequences are fully consistent.

In Figure 7 we showed both ApEn and SampEn for *m* = 2 and *r* = 0.2σ for the NYC temperatures considering sequences of ten years of data. We can see in the figure that the shape of the randomness is captured by both algorithms, with SampEn showing sharper differences. It is also important to notice that it is not possible to compare the absolute values of ApEn with those of SampEn, since the way of determining the randomness is different. Different values of *r* will provide different absolute values but, in general, the same shape.

### 4.3. ApEn vs SampEn

In this section, we compare the behavior of the two statistics using the sequence of data from 1 January 1869 to 31 December 1878 as an example. According to [31], SampEn is a more accurate measure because ApEn gives the data series a degree of regularity higher than the real value. However, SampEn is derived from Rényi entropy, meaning it is a lower bound of the real measure of information, and lower values indicate more regularity.

**Figure 9:**
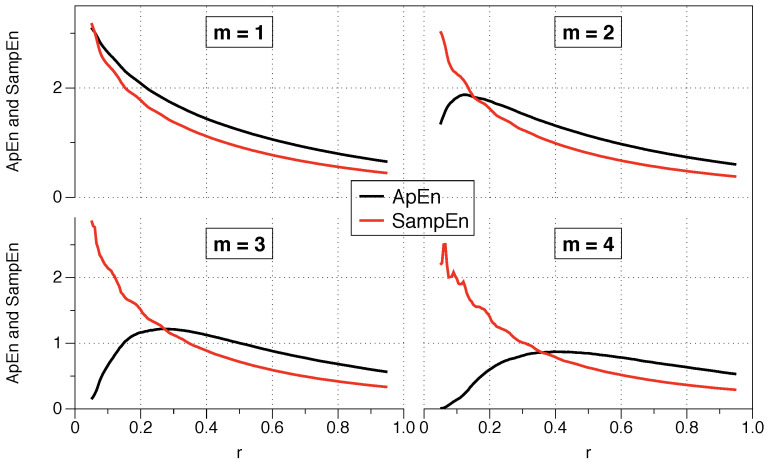
Dependency with *m* and *r* of SampEn and ApEn.

We start by analyzing the dependence of the two statistics on *m* and *r*. From Figure 9 we can obtain different conclusions. First, as the value of *m* increases, the statistics differ more, being very similar for *m* = 1 and differentiating their behavior for *m* = 4. Secondly, we see how SampEn is monotonically decreasing while ApEn presents a maximum. The appropriate behavior of a statistics which measures regularity is that of SampEn: when the value of *r* is small, there are very few matches in the patterns (since the condition is very restrictive), being the series very irregular at that moment, giving higher values of the statistics. The condition relaxes as *r* increases in value, and there are more matches, increasing the regularity (more predictable, lower values). That is the reason why ApEn is said to grant more regularity than the real value: if we choose the value of *r* before the maximum, we may be determining the randomness of the series incorrectly.

We make now a short analysis of the ApEn and SampEn equations shown in the previous chapter (Equation (27)). As we saw there, the fundamental differences between these two statistics were: that SampEn does not allow self-counting while ApEn does; that SampEn was of the form $\log(\sum_{i=1}^{N-m})$ while ApEn was $\sum_{i=1}^{N-m}\log$; and that ApEn included a prefactor $\frac{1}{N-m}$. We remember the equations here:

(29) $$\begin{array}{c} SampEn\left(m,r,N\right)=-\log\frac{\sum_{i=1}^{N-m}\sum_{j=1,j\neq i}^{N-m}\left[\mathrm{number}\;\mathrm{of}\;\mathrm{times}\;\mathrm{that}\;d[\right|x_{m+1}\left(j\right)-x_{m+1}\left(i\right)|]<r]}{\sum_{i=1}^{N-m}\sum_{j=1,j\neq i}^{N-m}\left[\mathrm{number}\;\mathrm{of}\;\mathrm{times}\;\mathrm{that}\;d[\right|x_{m}\left(j\right)-x_{m}\left(i\right)|]<r]}\\ ApEn\left(m,r,N\right)≃-\frac{1}{N-m}\sum_{i=1}^{N-m}\log\frac{\sum_{j=1}^{N-m}\left[\mathrm{number}\;\mathrm{of}\;\mathrm{times}\;\mathrm{that}\;d[\right|x_{m+1}\left(j\right)-x_{m+1}\left(i\right)|]<r]}{\sum_{j=1}^{N-m}\left[\mathrm{number}\;\mathrm{of}\;\mathrm{times}\;\mathrm{that}\;d[\right|x_{m}\left(j\right)-x_{m}\left(i\right)|]<r]}. \end{array}$$

In cases where *r* is small, we have seen that SampEn offered a higher value while ApEn incorrectly gave low values. A very low *r* is a restrictive situation in which it is difficult to find matches in the patterns, and the result should be that the series is very random (high values of the statistics). On the other hand, when *r* becomes larger, the condition relaxes, and the algorithm finds more patterns, increasing the number of times that the distance between coordinates is less than *r*, fulfilling the condition and providing lower values of the statistics. Let us suppose the limiting case in which this condition is met for all the patterns. In that case, the sum $\sum_{i=1}^{N-m}$ would become a product (N−m) (since $\sum_{i=1}^{N-m}a$ = (N−m)·a), and the previous equations would be:

(30) SampEn(m,r,N)=−log(N−m)∑j=1,j≠iN−m[numberoftimesthatd[|xm+1(j)−xm+1(i)|]<r](N−m)∑j=1,j≠iN−m[numberoftimesthatd[|xm(j)−xm(i)|]<r]ApEn(m,r,N)≃−1N−m(N−m)log∑j=1N−m[numberoftimesthatd[|xm+1(j)−xm+1(i)|]<r]∑j=1N−m[numberoftimesthatd[|xm(j)−xm(i)|]<r],

being the two statistics similar in this case, ApEn ≃ SampEn. That is the reason why both statistics coincide in Figure 1 of [31] for i.i.d. samples. On the other hand, for small *r* the differences between the two statistics are extensive, suggesting us that the different behavior is mainly a consequence of the self-counting introduced in ApEn. For ApEn, when *r* is very low, the template vector finds no coincidence except itself, so the probability (matches/possible vectors) is one, and the logarithm of that probability is zero, giving ApEn a low value which indicates regularity when in reality that situation should be considered as very random (there are no similar patterns). As we have discussed before, eliminating the self-counting in ApEn would lead to situations of log(0) and the statistic would be undefined.

**Figure 10:**
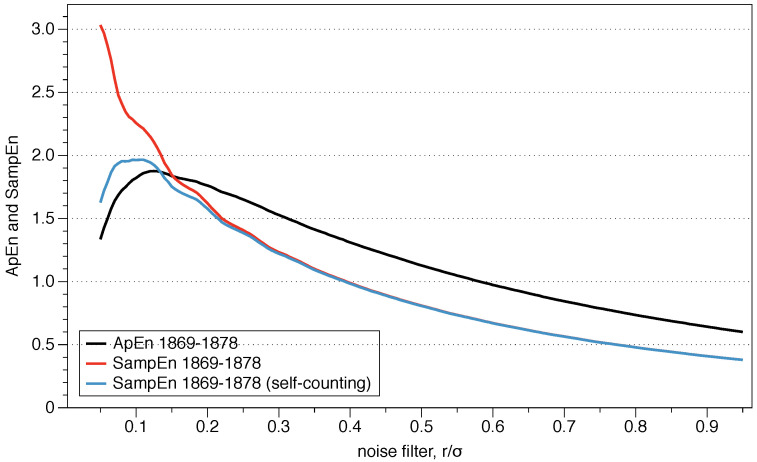
Behavior of SampEn when we allow self-counting.

However, we can try to analyze the effect of the bias by merely allowing self-counting in SampEn. Figure 10 shows the results of the analysis, where we observe that when the self-counting bias is allowed in SampEn, the behavior is very similar to that of ApEn. In the absence of such self-counting, the ApEn value would continue above SampEn (remember that $K_{2}<K_{1}$, i.e., SampEn would be a lower bound to the ApEn value, as in this example for r>0.16). Therefore, it is essential to use a value of *r* equal to or higher than the value at which ApEn finds its maximum to correctly determine the complexity of the series, since beyond that point the series are completely consistent.

## 5. Conclusions

The complexity of a series of data can be estimated by using algorithms such as ApEn or SampEn. The theoretical ideas behind them have roots in information theory and the concept of entropy, a magnitude which allows us to measure the amount of information in a mathematical way. Using the ideas of IT, researchers in Chaos Theory formulated different ways to determine the entropy with the intention to classify chaotic systems. However, even though those equations are useful for deterministic processes, small amounts of noise make them invalid for real series of data.

While the presented algorithms were initially developed for clinical research, they later spread to diverse fields such as neuroengineering [35], visual pattern recognition [36], neuroinformatics [37], ecology [38], psychiatry [39], electronics [40], voice recognition [41] or finance [42]. As their application is increasing lately, in this paper we have presented a clear path for understanding the logic behind ApEn and SampEn to help researchers understand their foundations and correct application.

In many situations, researchers make use of the presented algorithms without knowing the fundamentals behind them, which may lead to incorrect interpretations of the results. For example, a common misconception is that ApEn provides more regularity than the real value. However, this claim is only true if the selected value of *r* is before the maximum of ApEn; beyond that point, SampEn provides more regularity than the real value, due to its formulation based on Rényi entropy of order two.

Another clarification has been made to explain with an example the process of calculating ApEn. In order to have meaningful results, the comparison must be done between similar series (homogeneous processes) and usually with stationary measurements. We have also showed that the curvature of ApEn is due to the self-counting, which provides a more clear indication of how to select the noise filter *r*.

Finally, we provide simple source codes for the calculation of ApEn and SampEn in the Appendix A and Appendix B.

## Figures and Tables

**Table 1:** Probability table.

Block	x(1)	x(2)	x(3)	x(4)	x(5)	x(6)	x(7)	x(8)	x(9)	x(10)	x(11)
Possibles	2	3	3	3	3	3	3	3	3	3	2
Matches	2	3	3	3	3	3	3	3	3	3	2

## Abbreviations

The following abbreviations are used in this manuscript:

ApEn	approximate entropy
i.i.d.	independent and identically distributed
IT	information theory
MEP	maximum entropy principle
SampEn	sample entropy

## Appendix A. ApEn Code in R

The next algorithm is essentially the same one presented in [33]. Instead of FORTRAN, the algorithm has been translated to R, and we have avoided the use of “GO TO” functions, substituting those lines with nested loops to give a clearer understanding. It is important to notice that, as explained in the main text, this is an approximation of the ApEn statistics. See Equation (25) and the explanations above it.

1 # Step 1: read the data series

2     u <- read.csv("dataseries.csv")

3  

4 # Step 2: set the ApEn parameters m and r

5 #   optional: make the series stationary

6 # ------------------------------------------

7     u <- (u-mean(u))/sd(u)

8     NSUM <- length(u) - m

9  

10     m <- 2

11     Sigma = sd(u)

12     r <- 0.2∗Sigma

13  

14 # initiate variables to zero

15     pos <- rep(0,NSUM)

16     mat <- rep(0,NSUM)

17  

18     ratio <- 0

19     apen <- 0

20  

21 # ------- APEN CALCULATION BLOCK --------------

22  

23     for(i in 1:NSUM){            # Step 3: template vector

24  

25         posibles <- 0

26         matches <- 0

27  

28         for(j in 1:NSUM){        # Step 4: look for matches in the whole series

29  

30             for(k in 1:(m+1)){

31  

32                 # first possibility: it does not fit the condition for k < m

33                 if(k<m){

34                     dif <- abs(max(u[i+k-1] - u[j+k-1]))

35                     if(dif > r){

36                         break

37                     }

38                 }

39  

40                 # second possibility: check if it is a possible block or not, k = m

41                 if(k == m){

42                     dif2 <- abs(max(u[i+k-1] - u[j+k-1]))

43                     if(dif2 > r){

44                         break

45                     } else{

46                         posibles <- posibles + 1

47                         next

48                     }

49                 }

50  

51                 # third possibility: check if it is a match block or not, k = m+1

52                 if(k > m){

53                     dif3 <- abs(max(u[i+k-1] - u[j+k-1]))

54                     if(dif3 > r){

55                         break

56                     } else{

57                         matches <- matches + 1

58                         next

59                     }

60                 }

61  

62             }

63         }

64  

65         pos[i] <- posibles # save the probabilities for each template vector i

66         mat[i] <- matches # save the probabilities for each template vector i

67     }

68  

69  

70 # Step 5: phi functions

71 # --------------------

72     for(i in 1:NSUM){

73         ratio <- mat[i]/pos[i] # correlation integral

74         apen <- apen + log(ratio, base = exp(1)) # logarithmic sum of the phi functions

75     }

76  

77 # Step 6: ApEn calculation

78 # --------------------

79     apen = -1∗apen/NSUM

80     print(c(m,r/Sigma,apen))

81  

82 # ------- END APEN CALCULATION BLOCK --------------

## Appendix B. SampEn Code in R

1 # Step 1: read the data series

2     u <- read.csv("dataseries.csv")

3  

4 # Step 2: set the ApEn parameters m and r

5 #   optional: make the series stationary

6 # ------------------------------------------

7     u <- (u-mean(u))/sd(u)

8     NSUM <- length(u) - m

9  

10     m <- 2

11     Sigma = sd(u)

12     r <- 0.2∗Sigma

13  

14 # initiate variables to zero

15     pos <- rep(0,NSUM)

16     mat <- rep(0,NSUM)

17  

18     ratio <- 0

19     sampen <- 0

20  

21 # ------- SAMPEN CALCULATION BLOCK --------------

22  

23     for(i in 1:NSUM){            # Step 3: template vector

24  

25         posibles <- 0

26         matches <- 0

27  

28         for(j in 1:NSUM){        # Step 4: look for matches in the whole series

29  

30             if(j != i){ #Open condition: avoid self matches

31  

32             # first possibility: it does not fit the condition for k < m

33             for(k in 1:(m+1)){

34                 if(k<m){

35                         dif <- abs(max(u[i+k-1] - u[j+k-1]))

36                         if(dif > r){

37                           break

38                         }

39                 }

40  

41                 # second possibility: check if it is a possible block or not, k = m

42                 if(k == m){

43                         dif2 <- abs(max(u[i+k-1] - u[j+k-1]))

44                         if(dif2 > r){

45                             break

46                         } else{

47                             posibles <- posibles + 1

48                             next

49                         }

50                 }

51  

52                 # third possibility: check if it is a match block or not, k = m+1

53                 if(k > m){

54                         dif3 <- abs(max(u[i+k-1] - u[j+k-1]))

55                         if(dif3 > r){

56                             break

57                         } else{

58                             matches <- matches + 1

59                             next

60                         }

61                 }

62  

63               } #Close condition: avoid self-matches

64  

65             }

66         }

67  

68         pos[i] <- posibles # save the probabilities for each template vector i

69         mat[i] <- matches # save the probabilities for each template vector i

70     }

71  

72  

73 # Step 5: determine all total possibles and matches

74 # --------------------------------------------------

75     posibles <- 0

76     matches <- 0

77  

78 for(i in 1:NSUM){

79     posibles <- posibles + pos[i] #posibles

80     matches <- matches + mat[i] #matches

81 }

82  

83 # Step 6: SampEn calculation

84 # --------------------

85 ratio <- matches/posibles

86 sampen = -log(ratio, base = exp(1)) #sampen

87 print(c(r/Sigma,sampen))

88  

89 # ------- END SAMPEN CALCULATION BLOCK --------------
